# How will mosquitoes adapt to climate warming?

**DOI:** 10.7554/eLife.69630

**Published:** 2021-08-17

**Authors:** Lisa I Couper, Johannah E Farner, Jamie M Caldwell, Marissa L Childs, Mallory J Harris, Devin G Kirk, Nicole Nova, Marta Shocket, Eloise B Skinner, Lawrence H Uricchio, Moises Exposito-Alonso, Erin A Mordecai

**Affiliations:** 1 Department of Biology, Stanford University Stanford United States; 2 Department of Biology, University of Hawaii at Manoa Honolulu United States; 3 Emmett Interdisciplinary Program in Environment and Resources, Stanford University Stanford United States; 4 Department of Zoology, University of Toronto Toronto Canada; 5 Department of Ecology and Evolutionary Biology, University of California Los Angeles Los Angeles United States; 6 Environmental Futures Research Institute, Griffith University Brisbane Australia; 7 Department of Integrative Biology, University of California, Berkeley Berkeley United States; 8 Department of Plant Biology, Carnegie Institution for Science Stanford United States; Pennsylvania State University United States; Pennsylvania State University United States

**Keywords:** climate change, adaptation, evolutionary rescue, vector, mosquito, pest

## Abstract

The potential for adaptive evolution to enable species persistence under a changing climate is one of the most important questions for understanding impacts of future climate change. Climate adaptation may be particularly likely for short-lived ectotherms, including many pest, pathogen, and vector species. For these taxa, estimating climate adaptive potential is critical for accurate predictive modeling and public health preparedness. Here, we demonstrate how a simple theoretical framework used in conservation biology—evolutionary rescue models—can be used to investigate the potential for climate adaptation in these taxa, using mosquito thermal adaptation as a focal case. Synthesizing current evidence, we find that short mosquito generation times, high population growth rates, and strong temperature-imposed selection favor thermal adaptation. However, knowledge gaps about the extent of phenotypic and genotypic variation in thermal tolerance within mosquito populations, the environmental sensitivity of selection, and the role of phenotypic plasticity constrain our ability to make more precise estimates. We describe how common garden and selection experiments can be used to fill these data gaps. Lastly, we investigate the consequences of mosquito climate adaptation on disease transmission using *Aedes aegypti*-transmitted dengue virus in Northern Brazil as a case study. The approach outlined here can be applied to any disease vector or pest species and type of environmental change.

## Introduction

Climate change is expected to have major impacts on species distributions in coming decades, and predicting these impacts is an area of intense research interest. As their basic physiological and ecological traits depend heavily on temperature, climate impacts are expected to be particularly strong for ectotherms (Deutsch et al., 2008). This encompasses many taxa that threaten human health and well-being, including agricultural and forest pests, wildlife and plant pathogens, and disease vectors, for which accurately predicting distributions under climate change is critical for protecting human and animal health. Several prominent reviews have found that climate change is expected to increase, decrease, or, most commonly, shift the distributions of these taxa due to nonlinear and interactive effects of temperature and other climatic factors (Porter et al., 1991; Harvell et al., 2002; Deutsch et al., 2008; Rohr et al., 2011; Altizer et al., 2013; Lafferty and Mordecai, 2016; Pinsky et al., 2019; Lehmann et al., 2020; Rohr and Cohen, 2020). However, these predictions largely assume that species climate responses are fixed, ignoring the potential for adaptive responses.

Evidence of evolutionary adaptation to contemporary climate change has emerged for diverse taxa including mammals (Réale et al., 2003), fish (Kovach et al., 2012), plants (Franks et al., 2007; Exposito-Alonso et al., 2018), birds (Nussey et al., 2005; Karell et al., 2011), reptiles (Logan et al., 2018), and insects (Umina et al., 2005). However, while climate adaptation has typically been studied in the context of conservation biology, population genetics theory suggests that evolutionary adaptation is most likely for short-lived species with high population growth rates—properties of many pest, pathogen, and vector species (Lynch and Lande, 1993; Bürger and Lynch, 1995; Kingsolver, 2009). For several of these species, recent research demonstrates the potential for climate adaptation within a few decades. For example, in the European gypsy moth (*Lymantria dispar*)—one of the world’s most destructive forest pests (Montgomery and Wallner, 1988)—shifts in thermal tolerance were evident within 30 years of population expansion (Friedline et al., 2019). Similarly, in the Asian tiger mosquito (*Aedes albopictus*)—a vector of yellow fever, dengue, and chikungunya viruses—adaptive responses to novel temperature conditions were detected within 10–30 years of population expansions (Medley, 2010; Urbanski et al., 2012; Egizi et al., 2015; Medley et al., 2019). Despite this emerging evidence of the potential for rapid climate adaptation in pest and vector taxa, a general strategy for understanding and estimating their climate adaptive potential is lacking.

The potential for climate adaptation is a particularly important open question in mosquito-borne disease biology (Mordecai et al., 2019; Franklinos et al., 2019). Mosquito-borne diseases are a major public health burden, causing an estimated 500 million cases and millions of deaths globally each year (World Health Organization, 2014; World Health Organization, 2018). Environmental drivers of mosquito-borne disease transmission have been relatively well studied, and consistently highlight temperature—and by extension, climate warming—as a fundamental driver (Shragai et al., 2017; Mordecai et al., 2019; Franklinos et al., 2019; Shocket et al., 2021). Temperature influences mosquito-borne disease dynamics because it directly affects mosquito physiology, life cycles, behavior, and competence for disease transmission (Cator et al., 2020). For mosquitoes and other ectotherms, temperature has strong, nonlinear effects on traits such as survival and fecundity that lead to unimodal effects of temperature on fitness, where temperatures above and below intermediate thermal optima limit mosquito population growth (Huey and Stevenson, 1979; Huey and Berrigan, 2001; Angilletta, 2009; Amarasekare and Savage, 2012; Mordecai et al., 2019). Recent forecasts based on the unimodal relationship with temperature predict that in some areas where disease risk is currently high, future warming will decrease transmission risk as temperatures exceed mosquito thermal optima and limits (Gething et al., 2010; Ryan et al., 2015; Ryan et al., 2019; Mordecai et al., 2019; Mordecai et al., 2020). However, these predictions are likely to underestimate future disease risk if mosquitoes adapt to climate warming. As a result, estimating the potential for mosquito thermal adaptation is critical for more accurate predictive modeling.

Here, we outline a theoretical framework for investigating climate adaptive potential in ectotherm pests and pathogens, which draws from evolutionary rescue models typically used in conservation biology. We use mosquito adaptation to warming temperature as a focal case given the high global health burden of mosquito-borne disease and substantial recent research progress on mosquito thermal biology. In the following sections, we: (1) outline the theoretical framework and the specific parameters needed to estimate adaptive potential, (2) synthesize available information for mosquito thermal adaptation and identify key data gaps for predictive modeling, (3) highlight priorities and describe specific empirical approaches for filling these gaps, (4) explore the consequences of mosquito thermal adaptation on disease transmission, and (5) discuss the application of this framework to other vector and pest species. We focus here on adaptation to warming temperatures that exceed current mosquito thermal optima. We consider temperature in isolation despite its influence on relative humidity, which has strong impacts on mosquito population dynamics and host-seeking behavior and is predicted to shift with anthropogenic climate change. Several studies have investigated mosquito adaptation to desiccation (e.g. Kearney et al., 2009; Simard et al., 2009; Fouet et al., 2012), but few have investigated mosquito responses to simultaneous variation in temperature and humidity (but see Yamana and Eltahir, 2013; Yamana et al., 2016). Given this lack of empirical data, we focus on temperature specifically, but we discuss the inclusion of aridity in predictive modeling approaches (see ‘Climate factors currently limiting population persistence’). Similarly, we do not include non-climate factors such as biotic interactions, land use change, and human activities that may impact mosquito population persistence, because their effects on mosquito responses to temperature and thermal adaptive potential remain poorly understood. We discuss the adaptive potential of mosquitoes broadly, but our principal interest is in populations of major disease vector species (e.g. *Aedes aegypti, Ae. albopictus, Anopheles gambiae, Culex pipiens, Cx. quinquefasciatus*, which transmit dengue, chikungunya, Zika, and West Nile viruses, malaria, and other pathogens) and we discuss species-specific responses where possible. After presenting mosquito thermal adaptation as a focal case, we discuss how the approach we describe here can be applied to study the adaptive potential of any species in response to any specific environmental change.

### Framework for investigating climate adaptation

**Figure 1.**
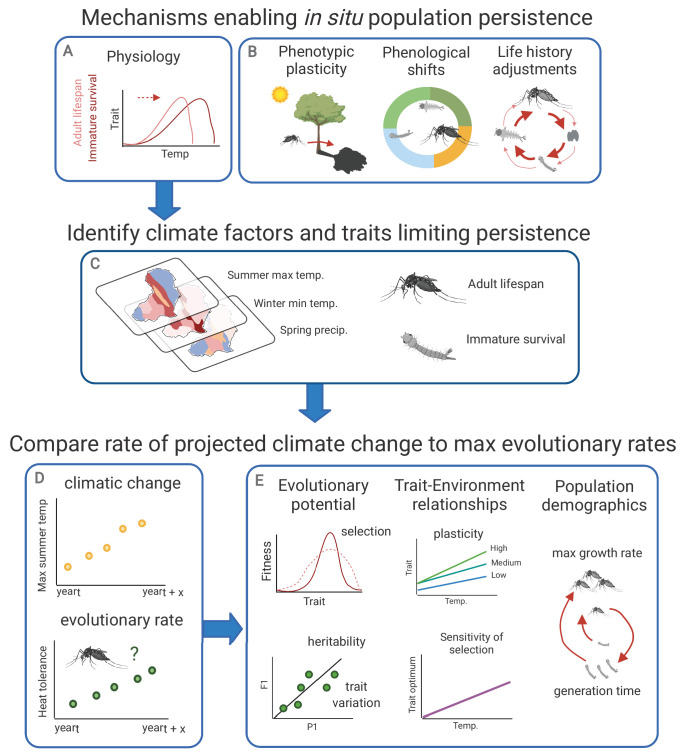
Framework for investigating climate adaptive potential. Several mechanisms may enable in situ population persistence (evolutionary adaptations in physiology, phenotypic plasticity, phenological shifts, and life history adjustments; panels A and B). Investigating the potential for evolutionary climate adaptation requires first identifying the climate factors and traits limiting population persistence (panel C), then comparing the rate of projected climatic change to potential evolutionary rates (panel D). Evolutionary rates can be estimated based on evolutionary potential (strength of selection, and heritability and variation in the trait of interest), population demographic characteristics (maximum growth rate and generation time), and trait – environment relationships (phenotypic plasticity and environmental sensitivity of selection) (panel E). In the strength of selection image (top left, panel E), the dashed and solid lines indicate the population before and after natural selection, respectively. In the heritability panel (bottom left), P1 and F1 denote the parental and offspring generations, respectively.

Species may respond to warming temperatures through three primary mechanisms: tracking suitable temperatures through range shifts, avoiding or temporarily coping with stressful temperatures through phenotypic plasticity (e.g. shifting biting activity to cooler times), and tolerating warming through genetic evolutionary adaptation (e.g. evolved shifts in thermal tolerance resulting from selection). Here, we focus on evolutionary adaptation as it would enable in situ population persistence under sustained environmental change and is currently the least well-understood climate response (Merilä and Hendry, 2014; Urban et al., 2016; González-Tokman et al., 2020). Investigating the potential for evolutionary climate adaptation requires identifying: (1) the climate factors currently limiting population persistence, (2) the most climate-sensitive and fitness-relevant traits, and (3) the potential evolutionary rates of these traits (Figure 1). We describe these factors further below, using mosquito thermal adaptation as a focal case.

### Climate factors currently limiting population persistence

Temperature fundamentally limits mosquito ranges and persistence through its influence on mosquito survival, development, and reproductive rates. However, the precise aspects of temperature that determine these limits remain unclear (Christophers, 1960; Yang et al., 2009; Brady et al., 2013; Shapiro et al., 2017; Tesla et al., 2018; Shocket et al., 2018; Mordecai et al., 2019). Temperature averages, variability, extremes, and interactions among these factors all impact ectotherm fitness (Nene et al., 2007; Lambrechts et al., 2011; Bozinovic et al., 2011; Kingsolver et al., 2013; Paaijmans et al., 2013; Blanford et al., 2013; Dowd et al., 2015; Buckley and Huey, 2016; Ma et al., 2021). The temperature variable that most strongly constrains persistence for a particular mosquito population will likely vary based on breeding and resting habitat preferences and by location, as thermal regimes can differ greatly between water sources used for oviposition, between indoor and outdoor environments, and by latitude and altitude (Paaijmans et al., 2008; Paaijmans et al., 2010; Paaijmans and Thomas, 2011). For example, changes in diurnal temperature variation may impose strong selection on East African *An. arabiensis* populations that primarily rest outdoors (Githeko et al., 1996), but have less impact on *An. gambiae* s.s and *An. funestus* populations that primarily rest indoors, where temperatures vary less drastically (Bødker et al., 2003; Minakawa et al., 2006). Similarly, endemic populations of a given species may experience vastly different climate regimes than invasive populations, leading to different constraints on their persistence (Moran and Alexander, 2014; Egizi et al., 2015). For example, *Aedes japonicus japonicus* populations in their native range in East Asia must contend with extreme cold winter temperatures, which they survive as diapausing eggs, while invasive populations in Hawaii and the southeastern U.S. experience warmer year-round temperatures (Reeves and Korecki, 2004; Larish and Savage, 2005; Kaufman and Fonseca, 2014). Based on evidence from other arthropod species, maximum annual temperature has been identified as the strongest driver of species extinctions (out of the 19 WorldClim Bioclimatic variables; Román-Palacios and Wiens, 2020), thus changes in maximum temperatures may exert the strongest selection pressure on mosquito populations near their warm range limits. As most temperature – trait responses are studied under constant temperatures (Deutsch et al., 2008; Angilletta, 2009; Paaijmans et al., 2013; Vasseur et al., 2014; Dowd et al., 2015; Buckley and Huey, 2016), we consider mosquito responses to increases in mean temperature as a focal example. Similarly, we consider the independent effects of temperature on mosquitoes despite its influence on moisture availability. Restrictions on moisture availability can readily be incorporated into predictive models by setting thresholds for annual precipitation or vegetative greenness (an indicator of local moisture availability; Suzuki et al., 2006), and masking out regions falling below these levels. For example, in estimating future temperature-based suitability of malaria transmission, Ryan et al., 2015 applied a threshold for Normalized Difference Vegetation Index (NDVI)—two consecutive months of NDVI above 0.125—and masked out all regions falling below this limit. In general, the framework we present can readily be applied to any specific measure of temperature or other environmental variable, such as temperature extremes, precipitation, wind patterns, land use change, and human activities (Reiter, 2001; Patz et al., 2008; Paaijmans and Thomas, 2011; Mordecai et al., 2019; Franklinos et al., 2019; Rocklöv and Dubrow, 2020).

### Mosquito traits

As selection acts on specific phenotypes, ‘adaptation’ here refers to a change in the thermal tolerance of a specific mosquito trait—an emergent property that reflects underlying physiological changes (e.g. changes in protein thermal sensitivity; Somero, 1995, Somero, 2003, Somero, 2010, González-Tokman et al., 2020). The critical traits to examine are those with the strongest climate sensitivity and the strongest impact on overall mosquito fitness. We hypothesize that mosquito life history traits with the lowest critical thermal maxima will experience the strongest thermal selection as environmental temperatures that exceed this limit have especially strong negative impacts on ectotherm fitness (Deutsch et al., 2008; Kingsolver et al., 2013). For several mosquito vector species, these traits are adult lifespan and fecundity, suggesting thermal selection may be strongest on the adult life stage (Figure 1; reviewed in Mordecai et al., 2019). However, whether these life history traits also pose the strongest constraints on mosquito fitness and persistence at high temperatures remains poorly understood because prior work has largely focused on mosquito traits related to disease transmission during the activity season, but there may be additional traits that help mosquitoes tolerate climate extremes (e.g. diapause, aestivation). For this reason, we consider several fitness-relevant mosquito life history traits (e.g. survival, development rates, and fecundity). Additionally, we consider various metrics that describe trait thermal tolerance (e.g. time to partial paralysis, known as ‘knockdown time’ at high temperatures; trait performances at high temperatures; and temperatures causing 50% sample mortality) because they may provide differing information on species adaptive potential (Hangartner and Hoffmann, 2016). The framework outlined here can be applied to any specific trait and measurement.

### Potential evolutionary rates of climate-sensitive traits

After identifying the climate factors and mosquito traits that limit population persistence, we can now compare their rates of change to predict whether populations can adapt apace with environmental change (Figure 1). To do so, we turn to evolutionary rescue models, which estimate the maximum rate of evolutionary change (i.e. adaptive genetic turnover) of a population and compare it to the projected rate of environmental change. Populations can persist only when their maximum sustainable evolutionary rate exceeds the required rate of evolution dictated by the environment (Bell and Gonzalez, 2009; Hoffmann and Sgrò, 2011; Gomulkiewicz and Shaw, 2013; Gonzalez et al., 2013; Carlson et al., 2014; Bell, 2017). Evolutionary rescue models explicitly model demographic rates and assume that populations are comprised of different genotypes with different reproductive advantages. As these models track population responses to sustained, directional environmental change, they are well-suited to estimating the potential for thermal adaptation in response to climate warming (Huey and Kingsolver, 1993; Bürger and Lynch, 1995; Chevin et al., 2010; Bay et al., 2017; Cotto et al., 2017; Diniz‐Filho et al., 2019), and have provided valuable estimates of climate adaptation potential across a range of taxa (Gienapp et al., 2013; Bush et al., 2016; Cotto et al., 2017; Diniz‐Filho et al., 2019). Even with incomplete or imprecise knowledge of all parameters, these models can place bounds on the climate response space to indicate where adaptation is highly unlikely and to inform future data collection efforts.

Here, we consider the analytic, quantitative-genetic evolutionary rescue model described by Chevin et al., 2010. This model estimates population adaptive potential under climate warming using (Box 1; Figure 1): (1) the maximum population growth rate under optimal conditions $(r_{max}$), (2) the population generation time (T), (3) the phenotypic variance in the trait of interest ${(\sigma }^{2})$, (4) the strength of selection imposed by temperature change (γ), (5) the trait heritability ($h^{2})$, (6) the degree of phenotypic plasticity in thermal tolerance (b), (7) how the trait optimum changes with temperature (i.e. environmental sensitivity of selection; B), and (8) the expected rate of temperature change during the time period $\left(\eta_{c}\right)$. Although the simplicity of this analytic evolutionary rescue model may constrain the accuracy of its projections, it illustrates the basic factors likely to affect population persistence, which we consider to be the minimum information needed to make initial predictions (see Appendix 1 for additional unmodeled factors and the ‘Priorities and approaches’ section for methods to incorporate additional complexity). We present the main findings from the available information below, including information from the closely related model organism *Drosophila* when little information is available for mosquitoes.

Box 1. Evolutionary rescue model formula (Chevin et al., 2010) and parameter definitions.

$$\eta_{c}=\sqrt{\frac{2r_{max}\gamma }{T}}\frac{h^{2}\sigma^{2}}{\left|B-b\right|}$$

T: **population generation time**: (for populations with discrete, non-overlapping generations), the mean time between reproduction in one cohort to reproduction in the successive cohort.

$r_{max}$: **maximum population growth rate:** the intrinsic rate of increase under optimal conditions (i.e. no intra- or inter-specific competition).

$\sigma^{2}$: **phenotypic variance**: the measured variance in the trait of interest.

$h^{2}$: **heritability**: the proportion of phenotypic variance in a trait attributable to additive genetic effects.

γ: **strength of selection:** the impact on fitness from deviations from the optimal trait value under a given environment. As in Kearney et al., 2009, a standardized version of selection strength can be approximated from temperature-dependent survival rates by:

$$i=2.2014-0.04884\;s+0.000558\;s^{2}-0.0000029\;s^{3}$$

where *s* is the percentage survival under a given environmental change, and *i* is given as the change in phenotype (in standard deviations) between the starting and selected populations (Falconer and Mackay, 1996; Matsumura et al., 2012).

b: **phenotypic plasticity:** the ability of individual genotypes to produce alternative phenotypes in different environments (Via et al., 1995). Here, plasticity encompasses thermal acclimation, dormancy and behavioral thermoregulation including shifts in mosquito biting, microhabitat usage, and oviposition sites and timing.

B: **environmental sensitivity of selection**: the change in the optimum phenotype with environmental change.

$\eta_{c}$: **maximum rate of environmental change:** the highest rate of sustained environmental change under which long-term population persistence is possible.

### Mosquito thermal adaptation: evidence and data gaps

#### Generation time (T) and maximum population growth rate ($r_{max}$)

Short generation times enable rapid evolutionary responses (Lynch and Lande, 1993; Bürger and Lynch, 1995), and high intrinsic population growth rates reduce the chance of extinction prior to adaptation (Bürger and Lynch, 1995; Orr and Unckless, 2008; Gomulkiewicz and Houle, 2009). The generally rapid life cycles and large population sizes of mosquitoes favor rapid evolution, but precise demographic estimates under natural conditions are unavailable for most species (but see Appendix 2—table 1 for growth rates and generation times for *Ae. aegypti, Anopheles spp., and Cx. pipiens*) and will vary with biotic and abiotic conditions. However, even high estimates of mosquito lifespans of approximately 3 months (Macdonald, 1952; Nayar and Sauerman, 1971; Papadopoulos et al., 2016; Joubert et al., 2016) are on par with or well below those of other species that have already demonstrated evolutionary responses to climate change (e.g. *Drosophila subobscura*, Rodríguez-Trelles and Rodríguez, 1998; Balanyá et al., 2006; *Tamiaschirus hudsonicus*, Réale et al., 2003; *Brassica rapa,* Franks et al., 2007, several bird species, Gienapp et al., 2007; *Cepaea nemoralis*, Ożgo and Schilthuizen, 2012; *Oncorhynchus gorbuscha*, Kovach et al., 2012). Further, high intrinsic population growth rates (r) of 0.19–0.38 per generation have been calculated for several major vector species (Appendix 2—table 1, Equation 1, Figure 1; Amarasekare and Savage, 2012; Johnson et al., 2015; Mordecai et al., 2017; Shocket et al., 2020) and census population size estimates on the order of 1,000–10,000 individuals have been found across studies of varying mosquito species and settings (Touré et al., 1998; Lehmann et al., 1998; Maciel-de-Freitas et al., 2008; Neira et al., 2014; Le Goff et al., 2019). Placing these mosquito results in context, a *Drosophila* modeling study showed that growth rates and population sizes in this range facilitated population persistence for over 300 generations under heat-knockdown selection (Willi and Hoffmann, 2009). Mosquito demographic characteristics therefore favor thermal adaptation.

### Variation in thermal tolerance ($\sigma^{2}$)

Higher genetically based variance in a trait results in higher rates of phenotypic evolution (Lande, 1976). While no studies (to our knowledge) have measured within-population variation in mosquito thermal tolerance, several studies have investigated variation between populations (Mogi, 1992; Reisen, 1995; Dodson et al., 2012; Vorhees et al., 2013; Ruybal et al., 2016; Chu et al., 2019). Overall, these studies find genetically based, but often trait-specific variation that did not always clearly support local thermal adaptation (i.e. a correlation between trait values and local climatic conditions; Appendix 3—table 1). Some studies have found thermal tolerance varying predictably with the population’s thermal environment of origin. For example, upper thermal limits of mosquito respiration and survival after heat shock were positively correlated with the temperature of origin for *Cx. tarsalis* and *An. gambiae,* respectively (Rocca et al., 2009; Vorhees et al., 2013). However, several other studies have found the opposite pattern (Ruybal et al., 2016), found minimal or no variation in thermal responses between populations (Dodson et al., 2012; Mogi, 1992), or found that certain populations had uniformly higher or lower trait performance at all experimental temperatures independent of their climate of origin (Ruybal et al., 2016; Reisen, 1995; Chu et al., 2019; Dodson et al., 2012). Taken together, mosquito populations do sometimes vary in their thermal performance, but there is no clear evidence for existing local thermal adaptation across temperature gradients of similar magnitude to those predicted by climate change over the next several decades. This may suggest either barriers to thermal adaptation or relatively weak selection on thermal performance (see ‘Strength of selection’ section). However, the lack of within-population sampling and/or idiosyncratic, trait-specific temperature relationships may have obscured true patterns of local adaptation (Bradshaw et al., 2000).

### Heritability of thermal tolerance ${(h}^{2})$

Higher heritability—the proportion of phenotypic variance in a population attributable to genetic effects—enables faster evolutionary rates because populations respond more efficiently to selection (Falconer and Mackay, 1996). To our knowledge, there are no estimates of the heritability of trait thermal tolerance for mosquitoes. However, evolutionary theory and empirical work in other ectotherm taxa suggest that thermal tolerance heritability is generally low. In particular, highly polygenic, complex, or environmentally-dependent traits—as expected for thermal tolerances—typically have low heritability (Bay et al., 2017). Supporting this expectation, a meta-analysis of heritability data for upper thermal limits in *Drosophila* resulted in an overall estimate of 0.28 (i.e. 28% of the populations phenotypic variance is due to genetic variance; Diamond, 2017), which is similar to heritability estimates for other *Drosophila* life history traits (average h^2^ = 0.26; Roff and Mousseau, 1987, Mousseau and Roff, 1987) and indicates moderately low heritability (but see Jenkins and Hoffmann, 1994, h^2^ = 0.5). However, more recent evidence suggests *Drosophila* can rapidly adapt to novel temperatures through multiple, alternative genetic pathways that lead to similar increases in thermal tolerance (i.e. ‘genetic redundancy’), challenging the notion that highly polygenic traits have low heritability (Barghi et al., 2019). In general, uncertainty surrounding the ecological relevance of laboratory measurements of insect thermal tolerance (Terblanche et al., 2007; Chown et al., 2009; Mitchell and Hoffmann, 2010) and the divergent evolutionary histories of Drosopholids and mosquitoes limit our understanding of thermal tolerance trait heritability in mosquitoes.

### Strength of selection (γ)

For heritable traits, stronger natural selection—the differential survival or reproduction of mosquitoes with different trait values—would lead to faster adaptive responses, despite causing high initial mortality (Box 1; Lynch and Lande, 1993; Hartl and Clark, 1997). Temperature-imposed selection on mosquitoes, which can be approximated from temperature-dependent survival rates (Box 1,γ; Falconer and Mackay, 1996), is likely to be strong. Upper thermal limits for adult and larval survival are as low as 32–38°C (reviewed in Mordecai et al., 2019), which many mosquito populations—particularly those in the tropics—already experience and will increasingly face in a warming climate (Deutsch et al., 2008). Further, steep declines in survival between thermal optima and critical limits have been observed across mosquito species (Focks et al., 1993; Alto and Juliano, 2001; Kamimura et al., 2002; Delatte et al., 2009; Muturi et al., 2011; Mordecai et al., 2019). This high selection pressure may facilitate mosquito adaptation, provided that heritable variation in trait thermal tolerance exists.

### Phenotypic plasticity (b)

Phenotypic plasticity—the ability of individual genotypes to produce varying phenotypes based on the environment (West-Eberhard, 2003)—provides an alternative mechanism for coping with climate change that is more rapid than evolutionary adaptation. However, because plasticity impedes natural selection on genetically based variation, it may ultimately inhibit population persistence under long-term directional change (Gienapp et al., 2008; Whitman and Agrawal, 2009; Chevin et al., 2010; Chevin et al., 2013; Merilä and Hendry, 2014). For mosquitoes, potentially important plastic responses include changes in activity patterns, biting behavior, or microhabitat selection, thermal acclimation, and initiation of dormancy, as reviewed below (and see Appendix 3—table 2). Phenotypic plasticity may itself vary across genotypes and thus could evolve in response to environmental change, but experimental evidence of the evolution of plasticity is lacking (Dewitt et al., 1998; Scheiner and Berrigan, 1998; Stinchcombe et al., 2004). Overall, mosquitoes possess a variety of potential plastic responses, but the capacity for these responses to increase thermal tolerance, their potential fitness costs, and how these plastic responses might interact with the process of evolutionary adaptation remain poorly understood. Below, we review current knowledge of different potential plastic responses.

### Behavioral thermoregulation

Larval and adult mosquitoes could temporarily cope with warming, particularly high-temperature extremes, through behavioral avoidance. In laboratory thermal preference studies, *Aedes, Anopheles*, and *Culex* spp. have demonstrated behavioral avoidance of high temperatures when exposed to a thermal gradient (Thomson, 1938; Blanford et al., 2009; Verhulst et al., 2020). In natural settings, several studies have shown shifts in the biting time or habitat selection of mosquitoes, particularly *An. gambiae*, seasonally or in response to insecticide spraying (Taylor, 1975; Reisen and Aslamkhan, 1978; Voorham, 2002; Pates and Curtis, 2005; Manda et al., 2011). While such behavioral shifts have not been conclusively linked to temperature, studies have found increasing usage of underground or shaded oviposition sites that was correlated with increasing temperature, and not associated with change in habitat availability or accompanied by genetic differentiation (*Ae. aegypti*, Somers et al., 2011; Chadee and Martinez, 2016). Similarly, larvae in permafrost regions were observed to rest in deeper, cooler pond water when surface water temperatures became exceptionally high (*Ae. communis*, a non-vector species; Haufe and Burgess, 1956). Seeking out and accessing cooler microclimates may buffer mosquitoes from warm temperature extremes, reducing mortality and decreasing the strength of selection. However, evidence for mosquito behavioral thermoregulation more generally remains limited (Paaijmans and Thomas, 2011; Waldock et al., 2013), and trade-offs in resource acquisition from restricted foraging and activity time, and a lack of readily available cooler microhabitats would constrain this behavior (Angilletta, 2009; Sears et al., 2016; Huey and Kingsolver, 2019). Conversely, the absence of evidence for this phenomenon may be due to measurement challenges associated with observing mosquitoes in the field (Paaijmans and Thomas, 2011).

### Thermal acclimation

Increases in thermal tolerance after exposure to warmer temperatures during development—a form of thermal acclimation—have been documented in several mosquito species (*An. albimanus*, Benedict et al., 1991; *An. arabiensis* and *An. funestus*, Lyons et al., 2012; *Cx. pipiens*, Gray, 2013; *Ae. aegypti,* Sivan et al., 2021). However, increases in thermal limits were typically minimal, suggesting a limited capacity for thermal acclimation to reduce mortality at high temperatures and enable population persistence. For example, the critical thermal limits of respirometry, motor function, or survival increased by less than 2°C for populations developing in 5°C warmer environments (Benedict et al., 1991; Lyons et al., 2012; Gray, 2013). Similarly, critical thermal maxima varied minimally with acclimation temperatures across a diverse range of over 200 ectotherm species (Gunderson and Stillman, 2015; Somero et al., 2016; Heerwaarden et al., 2016; Rohr et al., 2018).

### Dormancy

Temporarily unfavorable environmental conditions could be overcome through dormancy—the interruption or reduction of metabolic activity through diapause or quiescence—a response that has been demonstrated in all major vector species (reviewed in Diniz et al., 2017). Dry-season dormancy (i.e. aestivation) is likely one mechanism enabling *An. gambiae* and *An. coluzzi* to persist during the 3- to 6-month long dry season in the Sahel, as evidenced by very low population sizes during the dry season followed by rapid increases after the first rain (Lehmann et al., 2010; Lehmann et al., 2014; Adamou et al., 2011; Yaro et al., 2012; Dao et al., 2014). However, there are no known examples of dormancy mechanisms in ectotherms that respond solely to high temperatures, thus this may be an unlikely response for mosquitoes, particularly tropical species facing warming temperatures in humid environments.

### Environmental sensitivity of selection (B)

Environmental sensitivity of selection refers to how the optimum phenotype shifts with changes in the environment and is typically measured as the slope of the relationship between the optimal trait value and the environmental variable (e.g. the rate of change in the optimal upper thermal limit of adult life span against maximum summer temperature; Figure 1; Chevin et al., 2010, Chevin et al., 2015). A larger difference between the environmental sensitivity of selection and phenotypic plasticity (i.e. a greater deviation in the phenotype from the optimal value) necessitates faster adaptation (Chevin et al., 2010). For mosquitoes, as for nearly all other organisms, the environmental sensitivity of trait thermal tolerance has not been empirically measured (Chevin et al., 2010). However, across mosquito populations (Lyons et al., 2012; Vorhees et al., 2013) and species (Mordecai et al., 2019), upper thermal limits for most mosquito life history traits were less variable than lower thermal limits and optima. These patterns could reflect strong environmental sensitivity on lower thermal tolerance, intermediate sensitivity for the optimum, and weak sensitivity on upper thermal tolerance. However, it may more likely reflect evolutionary constraints on upper thermal tolerance (Kellermann et al., 2012; Hoffmann et al., 2013), or result from competing selection pressures, greater metabolic costs of heat versus cold tolerance, genetic constraints, or gene flow hindering thermal adaptation (Angilletta, 2009; Kristensen et al., 2016).

### Expected rate of environmental change ($\eta_{c}$)

Rates of environmental change will vary based on the specific temperature variable being considered (e.g. mean temperature of the hottest month or quarter, maximum temperature in the dry season, etc.), and depend on climate policy: projections of global mean annual surface temperatures in 2100 vary by over 3°C depending on the future climate scenario (Collins et al., 2013). However, while greater warming is projected for higher latitudes (IPCC, 2007), faster rates of adaptation may be necessary for tropical mosquito populations that already experience environmental temperatures close to their thermal optima and may experience large fitness costs under additional warming in the absence of adaptation (Deutsch et al., 2008; Somero, 2010; Ryan et al., 2015; Mordecai et al., 2019). For example, although *Ae. aegypti* and *Ae. albopictus* are highly adaptable and have expanded into temperate climates, they are vulnerable to climate warming in their tropical ranges as temperatures here are expected to exceed their thermal optima and upper thermal limits in coming decades (Ryan et al., 2019).

### Priorities and approaches for measuring adaptive potential

Addressing several key data and knowledge gaps will improve our ability to estimate mosquito adaptive potential (Table 1). As outlined above, there are virtually no estimates of the heritability of thermal tolerance traits, environmental sensitivity of selection, and within-population variation (and few estimates of between-population variation) in thermal tolerance for mosquitoes specifically. Additionally, we have a limited understanding of the role of phenotypic plasticity, particularly behavioral thermoregulation, in mosquito thermal tolerance. Although other parameters of the evolutionary rescue model (i.e. the strength of selection imposed by temperature change, mosquito generation time, and maximum population growth rate) are often not measured directly or precisely, we have relatively more information about these parameters and they are unlikely to be the primary constraints on evolutionary adaptation (see ‘Estimating evolutionary rates’). We therefore recommend that future research focus on measuring environmental sensitivity of selection, plasticity, and within-population variation and heritability in thermal tolerance. We discuss the most promising and feasible approaches for doing so below.

**Table 1.** State of knowledge on evolutionary rescue model parameters for mosquito and *Drosophila* species. Numbers correspond to references; colors correspond to data availability. Purple indicates that data for these parameters are readily available (but not for all species or contexts). Blue indicates that some data are available, but further collection is warranted. Green indicates that minimal or indirect data are available (e.g. dormancy mechanisms suspected based on rapid mosquito population increases following the dry season). Yellow indicates that no estimates are available on these parameters (to our knowledge). Measurements on variation in thermal tolerance are designated as ‘inter-population’ or ‘intra-population.’.

	Available data	State of knowledge	
	Some data		
	Minimal or indirect data		
	No data	Mosquitoes	*Drosophila*
Generation time		Mordecai et al., 2017; Johnson et al., 2015; Shocket et al., 2020	Crow and Chung, 1967; Lin et al., 2014; Fernández-Moreno et al., 2007; Ashburner, 1989; Emiljanowicz et al., 2014
Maximum population growth rate		Mordecai et al., 2017; Johnson et al., 2015; Shocket et al., 2020; Amarasekare and Savage, 2012	Siddiqui and Barlow, 1972; Emiljanowicz et al., 2014; Chiang and Hodson, 1950; Mueller and Ayala, 1981
Variation in thermal tolerance		[Inter-population variation] Ruybal et al., 2016; Dodson et al., 2012; Reisen, 1995; Mogi, 1992; Chu et al., 2019; Vorhees et al., 2013; Rocca et al., 2009	[Intra-population variation] Rolandi et al., 2018; Fallis et al., 2011 [Between-population variation] Sørensen et al., 2001; [Bibr bib235][Bibr bib106]; ; Rashkovetsky et al., 2006; Lockwood et al., 2018
Heritability			Mitchell and Hoffmann, 2010; Huey et al., 1992; Hangartner and Hoffmann, 2016; Jenkins and Hoffmann, 1994; McColl et al., 1996; Castañeda et al., 2019
Strength of selection		reviewed in Mordecai et al., 2019	Rezende et al., 2020; Huey et al., 1991; Huey et al., 1992; Loeschcke and Hoffmann, 2007
Phenotypic plasticity	Acclimation	Gray, 2013; Lyons et al., 2012; Benedict et al., 1991; Armbruster et al., 1999; Sivan et al., 2021	MacLean et al., 2019; Hoffmann and Watson, 1993; Sgrò et al., 2010; Overgaard et al., 2011; Berrigan and Hoffmann, 1998
	Behavioral thermo-regulation	Reisen and Aslamkhan, 1978; Voorham, 2002; Barrera et al., 2008; Haufe and Burgess, 1956; Verhulst et al., 2020; Blanford et al., 2013; Thomson, 1938	Castañeda et al., 2013; Dillon et al., 2009; MacLean et al., 2019; Huey and Pascual, 2009; Wang et al., 2008; ; Feder et al., 1997; Gibbs et al., 2003
	Dormancy	Dao et al., 2014; Lehmann et al., 2010; Adamou et al., 2011; Yaro et al., 2012	Tatar et al., 2001
Environmental sensitivity of selection			

Selection experiments are a powerful tool for investigating the evolution of complex traits (reviewed in Fuller et al., 2005; Garland and Rose, 2009; Swallow et al., 2009) that can be used to estimate several of the parameters in evolutionary rescue model parameters. In artificial selection experiments, where individuals are chosen to advance to the next generation based on their value for a particular trait (e.g. time to thermal knockdown), heritability can be measured as: $h^{2}=R/(i\sigma_{p})$ (Falconer and Mackay, 1996). Here, *R* is the mean difference in the trait between control and selected lines, $\sigma_{p}$ is the trait standard deviation in the control lines, and *i*, the intensity of selection, is determined based on what proportion of the population is selected each generation (see Box 1). In laboratory natural selection—in which the treatment conditions, rather than the researcher, impose the selection pressure—selection strength itself can be approximated based on the survival rates between generations held at specific temperatures (see Box 1). Both selection designs have been used extensively with model organisms such as *Drosophila* spp.*, Daphnia* spp., and *Escherichia coli* to measure changes in upper limits of trait thermal tolerance. While no thermal selection experiments have yet been published on mosquitoes (Dennington et al., in prep), several major vector species, including *Ae. aegypti* and *Cx. quinquefasciatus,* can be readily maintained and manipulated in the lab (Munstermann, 1997; Kauffman et al., 2017) and can therefore be used in experiments to obtain estimates of the heritability of thermal tolerance and the selection strength imposed by different temperature conditions.

Common garden experiments, where traits are measured for distinct populations or genotypes exposed to the same environmental conditions, enable measurement of nearly all rescue model parameters (Clausen et al., 1941; Merilä and Hendry, 2014; de Villemereuil et al., 2016; Berend et al., 2019). In mosquitoes, common garden experiments have been used to investigate variation in thermal tolerance between populations sampled across a thermal gradient (Mogi, 1992; Reisen, 1995; Rocca et al., 2009; Vorhees et al., 2013; Ruybal et al., 2016; Chu et al., 2019), but this approach could also be used to measure within-population variation if thermal tolerance traits were measured at the individual level. For example, larval development rates or adult survival time could be measured by tracking mosquitoes housed individually under different temperature conditions (as in Bedhomme et al., 2003). To measure genetically based variation in thermal tolerance, and to avoid confounding parental effects and thermal acclimation in the original environment, collected populations should be reared for at least one generation in the lab before experimentation. However, plasticity itself can be measured by, for example, varying larval rearing temperature (Dodson et al., 2012) or measuring thermoregulatory behavior as the trait of interest (e.g. Logan et al., 2018). Common garden experiments can also be used to measure the environmental sensitivity of selection if fitness is measured in addition to thermal tolerance traits (Chevin et al., 2010). Lastly, by tracking parentage and measuring thermal tolerance traits (e.g. by housing mating pairs of mosquitoes separately), the heritability of thermal tolerance can be measured based on the slope of the trait values of parent and offspring (Falconer and Mackay, 1996).

In addition to providing estimates of rescue model parameters, common garden experiments can be combined with genomic approaches to identify genetic variants associated with climate-adaptive traits (for examples in non-mosquito species, see De Kort et al., 2014; de Villemereuil et al., 2016; Exposito-Alonso et al., 2019; Capblancq et al., 2020). In the closest example of this approach in mosquito populations, the hypothesized thermo-adaptive role of a particular genotype (chromosomal inversion 2La) associated with aridity clines in Africa in *An. gambiae* (Coluzzi et al., 1979) was confirmed based on thermal tolerance experiments on the two genotypes (homokaryotypic populations 2La+ and 2La) (Rocca et al., 2009). In other taxa, common garden experiments have been combined with genome scans to quantify and predict climate-driven selection along the genome of the plant *Arabidopsis thaliana* (Exposito-Alonso et al., 2019), and to identify 162 candidate genes underlying climate adaptation in the harlequin fly *Chironomus riparius* (Waldvogel et al., 2018). In these studies, whole-genome sequencing would provide greater power to detect causal loci, thus this approach would be most feasible for mosquito species with available reference genomes, namely *Ae. aegypti* (e.g. Nene et al., 2007; Matthews et al., 2018), *Ae. albopictus* (Chen et al., 2015), *An. darlingi* (Marinotti et al., 2013), *An. gambiae* (Holt et al., 2002), and *An. stephensi* (Jiang et al., 2014).

Selection experiments and common garden experiments provide the means to obtain critical missing information on mosquito adaptive potential, but there are several challenges to these approaches. For any experimental test of adaptive potential, regardless of the methodology used, one must identify appropriate temperature treatments and assess thermal tolerance on the mosquito life history traits most relevant for fitness. Arbitrary choices for these details make it more difficult to extrapolate from these results to natural systems. Experiments commonly use treatments with constant temperatures above mean ambient temperatures. However, temperature minima or maxima, seasonal variability, and/or accumulated thermal stress may be more relevant to adaptive potential. For example, increases in minimum temperatures affect overnight recovery from heat stress in mosquitoes (Murdock et al., 2012; Bai et al., 2019). Further, given trade-offs in isolating the effect of temperature versus incorporating realistic ecological variation and in maximizing replication between versus within populations, no single study can definitively determine a species’ adaptive potential. As a first step, controlled and replicated lab studies measuring mosquito fitness (either directly or as a composite of individual life history traits) under realistic projected thermal regimes that incorporate natural diurnal variation in temperature, combined with genomics approaches, will greatly improve our understanding on current and potential mosquito thermal adaptation (Andriamifidy et al., 2019). Such studies will inform parameters of evolutionary rescue models and, more broadly, enable investigation of the dynamics and limits of thermal adaptation.

While these empirical approaches will address data gaps that we have emphasized within the evolutionary rescue framework (Table 1), the model itself (Chevin et al., 2010) has several important limitations. Notably, these include the lack of potential genotype-by-environment interactions in the expression of phenotypes, evolution in plasticity, gene flow, genetic correlations between traits associated with thermal tolerance, and demographic or environmental stochasticity (Chevin et al., 2010). These simplifying assumptions make the model tractable but may limit the accuracy of the predictions if these factors play a large role in adaptation. Adding complexity would require additional data collection and may make predictive models too computationally intensive to solve analytically but can be implemented through simulations (Bürger and Lynch, 1995). Several studies have effectively used simulations to incorporate environmental stochasticity (Ashander et al., 2016), demographic stochasticity (Martin et al., 2013), dispersal (Schiffers et al., 2013), carrying capacity (Bridle et al., 2010), and evolution in plasticity (Scheiner et al., 2017) into an evolutionary rescue model framework. Simulation results can be used to investigate transient evolutionary dynamics and can be compared with analytic results to determine the impact of these processes on evolutionary rescue. For example, Ashander et al., 2016 estimated population extinction risk using both analytic approximations and simulations to find that evolving plasticity only facilitated evolutionary rescue when the environmental change was sufficiently predictable. Using simulation to model more realistically complex evolutionary scenarios will likely be necessary when more precise forecasting is a priority, and is becoming a more approachable method through the availability of individual-based evolutionary simulation tools such as SLiM (Haller and Messer, 2017). For example, Matz et al., 2020 used the SLiM framework to estimate the adaptive potential of a coral metapopulation under varying levels of mutation, migration, and selection efficiency, enabling them to identify the main predictors of adaptive potential and the scenarios enabling long-term coral persistence.

### Consequences for disease transmission

Current modeling approaches for predicting mosquito-borne disease transmission under climate change do not incorporate evolutionary adaptation in mosquito thermal tolerance. In particular, several studies have used a temperature-dependent $R_{0}$ modeling approach (where $R_{0}$ is the average number of secondary cases that result from a single infected individual introduced into a fully susceptible population) to estimate transmission of mosquito-borne diseases including dengue, chikungunya, Zika, and malaria under projected temperature conditions (e.g. Ryan et al., 2015; Ryan et al., 2019; Mordecai et al., 2017; Tesla et al., 2018). These studies rely on relationships between temperature and mosquito life history traits previously measured in the lab and currently provide the best estimates of mosquito-borne disease transmission under climate warming. However, if mosquito thermal optima and upper thermal limits increase, these predictions would underestimate future disease risk.

To investigate the consequences of shifts in mosquito thermal limits on disease transmission predictions, we consider a case study using *Aedes aegypti*-transmitted dengue virus in Northern Brazil (Appendix 4). This region, which includes the North and Northeastern Brazilian macroregions, experiences approximately 250,000 dengue cases annually (National Notifiable Diseases Information System (SINAN), 2019), primarily transmitted by *Aedes aegypti* (Chouin-Carneiro and Barreto dos Santos, 2017). In the absence of mosquito thermal adaptation, Ryan et al., 2019 projected that year-round transmission suitability would decrease in this area by 2080 under an upper climate change scenario (representative concentration pathway (RCP) 8.5). We repeat the modeling approach used in this projection to examine the rate of evolutionary adaptation required by *Aedes aegypti* to maintain current levels of dengue transmission suitability (Appendix 4). We use the same temperature-dependent $R_{0}$ model to estimate the number of months per year where temperatures do not prevent dengue transmission (i.e. $R_{0}(T)$ > 0, as defined previously in Ryan et al., 2019) under current (2021) climate conditions and in 2080 under RCP 8.5. We then estimate the amount of evolutionary change in *Aedes aegypti* thermal limits necessary to maintain current levels of transmission suitability. We assume that adult fecundity is the mosquito trait under thermal selection as it has the lowest critical thermal maximum (34.61°C) of the *Ae. aegypti* and dengue virus life history traits and thus sets the warm temperature limit for dengue transmission (Mordecai et al., 2017; Mordecai et al., 2019). As in Ryan et al., 2019, we use mean monthly temperature when estimating temperature-based suitability for transmission, although this is not necessarily the climate factor that most strongly limits *Ae. aegypti* persistence in this region.

**Figure 2.**
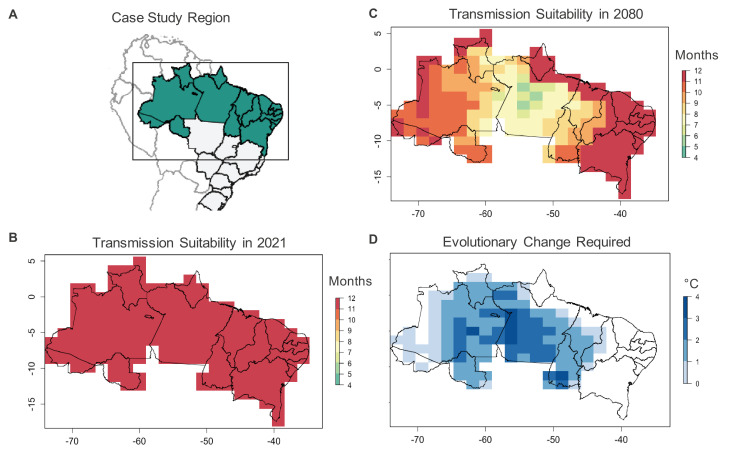
Case study on *Ae. aegypti*-transmitted dengue suitability. Under current conditions, monthly dengue transmission suitability (i.e., $R_{0}(T)$ > 0) based on mean monthly temperatures is high throughout Northern Brazil (A, B). Transmission suitability is projected to decline by 2080 under the RCP 8.5 climate scenario (C), as temperatures exceed mosquito upper thermal limits. To maintain current monthly transmission suitability under temperatures projected for 2080, evolutionary change, in the form of an increased critical thermal maximum of *Ae. aegypti* fecundity (D) may be required, with greater evolutionary change required in areas with greater projected warming.

We find that in the absence of thermal adaptation in *Ae. aegypti* fecundity, the average number of months per year with suitable temperatures for dengue transmission in Northern Brazil would decrease from 12.0 in 2021 to 10.3 in 2080 (Figure 2). To maintain current (2021) levels of dengue transmission suitability under 2080 temperatures, the critical thermal maximum of *Ae. aegypti* fecundity would need to increase by an average of 1.57°C in this time period, or roughly 0.03°C/year. This evolutionary rate is on par with sustainable evolutionary rates estimated for other taxa and traits in the face of climate warming (e.g. great tit breeding time: 0.03–0.10°C/year; Gienapp et al., 2013). However, determining whether this is a plausible rate of evolutionary change in fecundity for *Ae. aegypti* in this region will require collecting missing information on the evolutionary rescue model parameters (Table 1) through the empirical approaches described above. In this case study, estimating the thermal adaptive potential of *Ae. aegypti* fecundity would help determine whether or not the dengue transmission season may decrease by nearly 2 months in a region containing approximately 69 million people (IBGE, 2010).

As explored in the case study, maintaining disease transmission under climate warming may require evolved increases in mosquito upper thermal limits. However, such evolutionary shifts could maintain, increase, or decrease transmission depending on whether they are accompanied by shifts in lower thermal limits, on the strength of thermodynamic constraints, and on genetic correlations between traits. In the absence of other changes to thermal performance, upward shifts in thermal limits could maintain current levels of disease transmission under rising temperatures, particularly if lower temperatures are infrequently experienced. However, disease transmission may increase if peak performances for mosquito traits like fecundity and biting rate increase with their thermal optima. This is an expectation of the ‘hotter-is-better’ hypothesis, but how the shape of thermal performance curves evolves is a point of ongoing debate and empirical uncertainty (Angilletta et al., 2010; Latimer et al., 2011; Kontopoulos et al., 2020). Regardless, genetic correlations between mosquito traits under direct selection and other traits that may impact disease transmission (e.g., development time and immunocompetence, as observed in *Ae. aegypti*; Koella and Boëte, 2002) could still constrain mosquito-borne disease transmission under thermal adaptation (Lande and Arnold, 1983).

Mosquitoes, like other ectotherms, may cope with warming temperatures through a variety of other mechanisms besides shifts in thermal physiology, such as accelerated life cycles, phenological shifts, and behavioral thermoregulation, with varying consequences for disease transmission (Huey and Kingsolver, 1993; Bradshaw et al., 2000; Stearns et al., 2000; Angilletta et al., 2003; Waldvogel et al., 2020). Evolved increases in life cycle speed can mitigate increases in daily mortality rates, and were suggested to occur in *Anopheles* spp. in response to vector control interventions (Ferguson et al., 2012). Adult mosquito longevity is already the main limitation on transmission near upper thermal limits for many major mosquito-borne diseases (Mordecai et al., 2019). Further reductions in adult lifespan could cause large declines in transmission for pathogens with longer incubation periods. In particular, transmission of malaria parasites, which have a minimum incubation period of approximately nine days (Paaijmans et al., 2012; Blanford et al., 2013), may be more negatively impacted under shortened mosquito lifespans than viral pathogens such as dengue virus and chikungunya virus, which have generally faster incubation periods—as low as three to five days at temperatures above 30°C (Tjaden et al., 2013; Rudolph et al., 2014; Mordecai et al., 2019; Winokur et al., 2020). The implications of warming-driven life cycle adaptation therefore depend on the interaction between vector and pathogen traits, which vary across species and environments.

Behavioral thermoregulation and phenological shifts could increase, maintain, or decrease disease transmission, primarily depending on how these shifts impact mosquito – human contact rates and the effectiveness of vector control activities (Ferreira et al., 2017). For example, if rising temperatures promote shifts in biting activity towards the cooler, night-time hours when humans are more likely to be protected by bed nets, disease transmission may be reduced (Taylor, 1975; Pates and Curtis, 2005; Moiroux et al., 2012; Thomsen et al., 2017; Carrasco et al., 2019). However, in the absence of vector control, shifts towards night-time biting, as well as thermoregulatory shifts favoring indoor versus outdoor biting, could increase mosquito – human contact rates and transmission (Takken, 2002). Similarly, phenological shifts in mosquito activity could lead to changes in the length or timing of disease transmission, potentially maintaining, increasing, or decreasing disease transmission. For example, increasing monthly mean temperatures in portions of California have effectively doubled the potential transmission season of St. Louis encephalitis virus, such that elderly persons traveling to California for the winter are newly at risk (Patz and Reisen, 2001). Failing to account for phenological shifts in mosquito activity may render vector control programs less effective at reducing mosquito populations and disease transmission. In general, the impact of mosquito thermal adaptation on disease transmission will vary based on the mechanism of thermal adaptation, making identifying what adaptive strategies are most likely in different contexts a priority for future research.

### Applications to other vector and pest taxa

The same properties favoring mosquito thermal adaptation—short generation times, high population growth rates, and strong climate sensitivity—apply to many insect taxa that threaten human, animal, and plant health. These include vectors of major human, wildlife, and plant disease (e.g., species of tsetse flies, biting midges, psyllids, and aphids), as well as pests of crops and forest resources (e.g., species of beetles, moths, fruit flies, and fire ants). Despite the substantial societal cost adaptation in pest and disease vector species could impose, their potential to adapt to climate change remains poorly understood. This remains challenging to predict given the many determinants of evolutionary rates, incomplete data on these determinants for most taxa, and the inability to perform a single, conclusive experiment.

Drawing from conservation biology techniques used to study climate adaptive potentials in threatened and endangered species, we have outlined a framework and empirical approaches for investigating mosquito thermal adaptation that can be applied to any vector or pest species and type of environmental change. For example, in the Eastern U.S., range retractions of the invasive European gypsy moth have been linked to the duration of exposure above the optimal temperature for larval and pupal development (28°C; Tobin et al., 2014). Further, a recent common garden experiment found moth populations at the southern range edge, which experience the strongest thermal selection, have higher thermal tolerance in egg hatching, highlighting the potential for adaptive evolution in this species (Faske et al., 2019). The climate adaptive potential of this species could thus be estimated by comparing the potential evolutionary rates of thermal limits in immature development with projected rates of warming in this region. More precisely estimating the thermal adaptive potential of this pest species would enable forest management personnel to tailor intervention and control strategies in the face of ongoing warming. Similarly, estimates of mosquito thermal adaptive potential would enable vector control personnel to better target surveillance and insecticide applications to the appropriate locations and seasons of mosquito activity. More broadly, understanding and estimating the potential for climate adaptation in taxa of concern to human health is critical for accurately predicting and preparing for their persistence or shifts in their distributions under climate change.

### Conclusion: How will mosquitoes adapt to climate warming?

Our synthesis makes clear that some general aspects of mosquito demographics and strong temperature-imposed selection may facilitate rapid evolution and adaptation to climate warming. In particular, typical mosquito generation times and population growth rates are on par with those of species that have already demonstrated evolutionary responses to climate change. Further, the steep declines in survival at temperatures exceeding mosquito thermal optima, as well as evidence of some population-level variation in mosquito temperature-trait responses, indicate that the selective pressures and raw genetic material necessary for evolutionary adaptation exist. However, making more accurate predictions about mosquito persistence and adaptation under climate warming will require identifying: (1) which life history traits experience the strongest thermal selection for a particular mosquito population, (2) how the optimal trait thermal tolerance varies with environmental temperature, (3) the extent of heritability and within-population variation in trait thermal tolerance, and (4) the role of phenotypic plasticity (particularly behavioral thermoregulation) in evolutionary adaptation and persistence. Empirical approaches such as common garden or selection experiments to obtain multiple pieces of missing information at once and leveraging information from related taxa where applicable can be used to address these key knowledge gaps. This would enable better estimates of mosquito adaptive potential and its implications for the future of mosquito-borne disease in a warming climate.

### Data and code accessibility statement

All data and code supporting the results are either referenced in text, or available in the Appendices or on Github (https://github.com/lcouper/MosquitoAdaptationCaseStudy; copy archived at swh:1:rev:19a0d661adb3c0079bd5631be757cc1f255a854a, Couper, 2021).

## Figures and Tables

**Appendix 2—figure 1.**
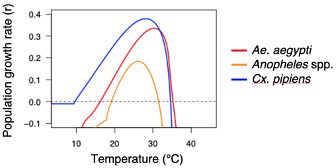
Population growth rate as a function of temperature for vector species listed in Appendix 2—table 1.

## Data Availability

All data and code supporting the results are either referenced in text, or available in the Appendices or on Github (https://github.com/lcouper/MosquitoAdaptationCaseStudy; copy archived at https://archive.softwareheritage.org/swh:1:rev:19a0d661adb3c0079bd5631be757cc1f255a854a).

## Appendix 1

Additional factors influencing population persistence In addition to the eight parameters in the Chevin et al., 2010 evolutionary rescue model (main text, Box 1), other factors influence a population’s potential for thermal adaptation. The starting population size informs the probability of obtaining rescue variants, and thus the probability of adaptation (Orr and Unckless, 2008; Bell, 2013; Martin et al., 2013; Carlson et al., 2014). The degree of stochasticity in temperature change influences an organism's ability to respond via plastic changes (Manenti et al., 2014; Catullo et al., 2015). The breadth of thermal performance and the genetic architecture of thermal tolerance inform the strength of selection (Huey and Kingsolver, 1993; Kopp and Matuszewski, 2014). Rates of gene flow and dispersal may hinder local adaptation due to the influx of maladapted genes but may promote evolutionary rescue through increasing genetic variation and bolstering small population sizes (Garant et al., 2007; Baskett and Gomulkiewicz, 2011; Kirkpatrick and Peischl, 2013; Schiffers et al., 2013; Bourne et al., 2014; Carlson et al., 2014). The degree of density dependence determines the strength of genetic bottlenecks (Nordstrom et al., 2020). Biotic interactions such as interspecific competition or predation may alter selection pressures (Culler et al., 2015), constraining rates of adaptation (Barbour et al., 2020), or limit population persistence despite sufficient rates of adaptation (Angilletta, 2009; Carlson et al., 2014; Johnson et al., 2019; Huey and Kingsolver, 2019). Fitness costs of phenotypic plasticity, such as reduced foraging associated with behavioral thermoregulation or energetic costs associated with maintaining the physiological machinery for acclimation may constrain levels of plasticity (Dewitt et al., 1998; Angilletta, 2009; Chevin et al., 2010).

## Appendix 2

Methods for calculating mosquito population growth rate The formula for population growth rate (*r*) as a function of temperature (*T*) is derived from the Euler-Lotka equation (Amarasekare and Savage, 2012) as follows:

(S1) $$r(T)=-\mu (T)+MDR(T)W\left(\frac{E(T)e^{\mu (T)-\frac{\mu_{j}(T)}{MDR(T)}}}{MDR(T)}\right)$$

Population growth rate is a function of adult mortality (µ), mosquito development rate (*MDR*), fecundity (*E*), and juvenile mortality (µ*_j_*). W is the upper branch of the Lambert function.

**Appendix 2—table 1.** Measurements of mosquito demographic rates for major mosquito vector species. Maximum population growth rates (r) were calculated using trait thermal responses from the references cited below and Equation S1 (Amarasekare and Savage, 2012). The temperature at which the maximum growth rate occurs, and the upper thermal limit for population growth (i.e., r = 0) are provided. The generation time is calculated as the sum of the immature development time, the gonotrophic period, and a minimum estimate of the host-blood meal and egg-laying time (4 days). We report the minimum generation time based on temperature.

Species	Max growth rate (r)	Max growth rate temperature	Upper thermal limit for growth rate	Minimum generation time (days)	Reference
*Ae. aegypti*	0.335	30.3°C	35.3°C	14	Mordecai et al., 2017
*Anopheles* spp.	0.187	26.2°C	31.6°C	17	Johnson et al., 2015
*Cx. pipiens*	0.379	28.1°C	34.6°C	17	Shocket et al., 2020

## Appendix 3

**Appendix 3—table 1.** Measurements of between-population variation in mosquito thermal tolerance. ‘Evidence of local thermal adaptation’ refers to measurements where populations from warmer source environments had higher thermal tolerance than those from cooler environments.

Species	Variation in source thermal environment	Thermal tolerance measurement	Evidence of local thermal adaptation?	Main finding	Reference
*Cx. pipiens*	~3°C in mean summer temperature	larval survival	no	Population from the coolest environment had the lowest survival at all temperatures	Ruybal et al., 2016
		adult survival	no	Population from the coolest environment had the lowest survival at cool temperatures, but highest survival at the warmest temperature	
		development rate	no	Population from the warmest environment had the highest development rate at all temperatures	
		biting rate	no	Population rank order varied with temperature	
*An. darlingi*	~7, 6, 13°C in annual mean, min, and max temperature, respectively	adult lifespan	no	Population rank order varied with temperature	Chu et al., 2019
		larval development	no	Population from the highest minimum temperature environment developed faster at all temperatures	
		wing length	no	Population from the coolest environment had the longest wing length at all temperatures	
*Cx. tarsalis*	~5, 6, 15°C in mean daily, mean daily max, and max recorded temperature (in summer)	metabolic activity	yes	Critical thermal limits correlated positively with mean daily max temperature of source environment (but not with mean daily or max recorded temperature)	Vorhees et al., 2013
*Cx. tarsalis*	Unspecified. Populations reared from two sites in CA, USA	larval development rate	no	Minimal variation between populations	Dodson et al., 2012
		% larval survival	no	Variation in survival that was strongest at the high temperature extreme	
		pupal development rate	no	Minimal variation between populations	
		% pupal survival	no	Variation in survival that was strongest at the high temperature extreme	
		wing length	no	No variation between populations	
*Cx. tarsalis*	~3°C difference in annual mean temperature	immature development rate	no	Population from warmer environment developed more quickly at all temperatures	Reisen, 1995
		development rate	no	Population from warmer environment developed more quickly at all temperatures	
		adult lifespan	no	Population from warmer environment had higher survival at intermediate, but not extreme temperatures	
*Cx. quinque-fasciastus*	Unspecified. Populations reared from sites in New Zealand, Fiji, and Japan	larval development	no	No variation between populations	Mogi, 1992
		adult emergence rate	no	No variation between populations	
		biting rate	no	Population rank order varied with temperature	
		ovariole numbers	no	Population from the intermediate environment had the greatest number of ovarioles at all temperatures	
		egg maturation	no	Minimal variation between populations

**Appendix 3—table 2.** Measurements of phenotypic plasticity in mosquito thermal tolerance, categorized as thermal acclimation, behavioral thermoregulation, and aestivation (see main text, ‘Phenotypic plasticity’).

Form	Species	Main finding	Reference
Thermal acclimation	*Cx. pipiens*	Critical thermal maxima increased 1°C when developed at 26°C versus 18°C	Gray, 2013
Thermal acclimation	*An. arabiensis* and *An. funestus*	Little variation in critical thermal maxima (typically increased by <2°C) after thermal acclimation	Lyons et al., 2012
Thermal acclimation	*An. albimanus*	Heat tolerance increased with warming developmental temperatures and with a prior heat shock exposure, but mosquitoes from all treatments died at 40–43°C	Benedict et al., 1991
Thermal acclimation	*W. smithii*	Larval and adult survival after heat shock increased ~0–30% in populations subjected to fluctuating hot/cold temperatures during development	Armbruster et al., 1999
Thermal acclimation	*Ae. aegypti*	Larvae pre-acclimated to warmer temperatures (37–39°C) survived longer at higher temperature extremes (43–45°C)	Sivan et al., 2021
Behavioral thermoregulation	*Anopheles* sp.	Observed seasonal shifts in feeding time, with biting occurring at dusk in cooler times of the year and late at night during warmer times	Reisen and Aslamkhan, 1978
Behavioral thermoregulation	*An. darlingi*	Observed correlation between time of year and crepuscular biting rates, and high within-population variation in biting time	Voorham, 2002
Behavioral thermoregulation	*Ae. communis*	Observed larvae resting in deeper, cooler waters when surface water temperatures became exceptionally high	Haufe and Burgess, 1956
Behavioral thermoregulation	*Ae. aegypti, Ae. japonicus*	Observed preference for 30°C when exposed to thermal gradient of 30–45°C in laboratory trials	Verhulst et al., 2020
Behavioral thermoregulation	*An. stephensi*	Observed preference for resting at ~26°C when exposed to thermal gradient of 14–38°C in laboratory trials	Blanford et al., 2009
Behavioral thermoregulation	*Cx. fatigans*	Observed avoidance of high temperatures when exposed to thermal gradient of 25–30°C in laboratory trials	Thomson, 1938
Aestivation	*An. gambiae, An. coluzzii*	Lower reproductive rates during the 3–6 month dry period were followed by rapidly rebounding population sizes after the first rain, suggesting persistence through aestivation	Yaro et al., 2012, Dao et al., 2014

## Appendix 4

Methods for ‘Consequences for disease transmission’ case study: Aedes aegypti-transmitted dengue virus in Northern Brazil Case study context In this case study, we estimate the monthly temperature suitability for dengue virus transmission by *Aedes aegypti* in Northern Brazil. This includes the North and Northeast macroregions, with a combined population size of approximately 69 million people (Instituto Brasileiro de Geografia e Estatistica and população, 2016) and 250,000 probable dengue cases per year (National Notifiable Diseases Information System (SINAN), 2019). Based on model projections that do not incorporate mosquito thermal adaptation (Ryan et al., 2019), *Ae. aegypti*-transmitted dengue suitability is expected to decline in this region by 2080 under an upper climate change scenario (RCP 8.5) as warming temperatures exceed mosquito upper thermal limits. Thermal adaptation by *Ae. aegypti* in this region could enable dengue transmission suitability to be maintained. Modeling approach We use a temperature-dependent model of $R_{0}$ —the number of secondary infections expected from a single infected individual introduced into a fully susceptible population—using the following expression (Mordecai et al., 2013):

(S2) $$R_{0}\left(T\right)=\sqrt{\frac{a{\left(T\right)}^{2}b\left(T\right)c\left(T\right)e^{-\frac{\mu \left(T\right)}{PDR\left(T\right)}}EFD\left(T\right)p_{EA}\left(T\right)MDR\left(T\right)}{Nr\mu {\left(T\right)}^{3}}}$$

where (*T*) indicates a temperature-dependent trait, *a* is the mosquito biting rate, *b* is the proportion of infectious mosquito bites resulting in infected humans, *c* is the proportion of bites on infected humans resulting in infected mosquitoes, µ is the adult mosquito mortality rate (here calculated as 1/ lifespan), *PDR* is the parasite development rate, *EFD* is the number of eggs laid per female per day, $p_{EA}$ is the mosquito egg-to-adulthood survival probability, *MDR* is the sub-adult mosquito development rate, *N* is the density of humans, and *r* is the human recovery rate. We use the data compiled by Mordecai et al., 2017 on the temperature-dependence of *Aedes aegypti* and dengue virus traits involved in transmission to parameterize the model. We use this model to estimate the number of months per year in which temperatures are suitable for dengue transmission (i.e., the number of months in which $R_{0}\left(T\right)>0$), a conservative threshold that defines the range of temperatures at which dengue transmission is not prohibited (Ryan et al., 2019). We first estimate the number of suitable months for transmission under current (2021) and future (2080) temperatures assuming no mosquito adaptation. We then estimate the extent of mosquito thermal adaptation needed to maintain current levels of transmission suitability under future temperatures. We assume adult fecundity to be the life history trait under thermal selection as it has lowest critical thermal maximum ($CT_{max}$; 34.61°C) of the *Ae. aegypti* and dengue virus life history traits (MacLean et al., 2019) and is thus the trait that determines the maximum temperature for dengue transmission. Current mean monthly temperatures do not exceed the ${CT}_{max}$ for fecundity at any location within Northern Brazil in 2021. By 2080 under RCP 8.5, mean monthly temperatures are often expected to exceed ${CT}_{max}$, indicating temperatures are projected to be unsuitable for dengue transmission (i.e., $R_{0}\left(T\right)=0$). We ask: What degree of increase in the ${CT}_{max}$ of *Ae. aegypti* fecundity would enable current monthly transmission suitability to be maintained in 2080? In this example, we consider fecundity as the only trait under thermal selection, although future temperatures may exceed the ${CT}_{max}$ of other *Ae. aegypti* and dengue virus life history traits, and there may be genetic correlations between fecundity and mosquito survival and development. Climate data For consistency with Ryan et al., 2019, we use mean monthly temperature when estimating $R_{0}\left(T\right).$ Other climate variables such as diurnal temperature variation or monthly precipitation may limit future *Ae. aegypti* persistence in this region, but their effects on mosquito life history traits are less well understood than those of mean temperature, and thus they are not considered here for simplicity. Regions in which extreme drought or other limiting climate conditions are projected could be masked from the analyses as in Ryan et al., 2015 for more precise estimates. For current and projected mean monthly temperature data, we used the Hadley general circulation model (GCM) (HadGEM2-ES), which has high performance in Brazil and is the most commonly used GCM (Ryan et al., 2019; Almagro et al., 2020). Data were accessed through the Earth System Grid Federation. We used temperature projections made under representative concentration pathway (RCP) 8.5 for consistency with Ryan et al., 2019. RCP 8.5 is considered a ‘business-as-usual’ fossil fuel emissions scenario and corresponds to an 8.5 ${W/}_{m^{2}}$ increase in solar radiation by 2100 (Riahi et al., 2011). All climate analyses and mapping were conducted in R version 4.0.2. The R code and climate data files used in the analysis are available on Github (https://github.com/lcouper/MosquitoAdaptationCaseStudy).

## References

[bib1] Adamou A, Dao A, Timbine S, Kassogué Y, Yaro AS, Diallo M, Traoré SF, Huestis DL, Lehmann T (2011). The contribution of aestivating mosquitoes to the persistence of *anopheles gambiae* in the Sahel. Malaria Journal.

[bib2] Almagro A, Oliveira PTS, Rosolem R, Hagemann S, Nobre CA (2020). Performance evaluation of eta/HadGEM2-ES and eta/MIROC5 precipitation simulations over Brazil. Atmospheric Research.

[bib3] Altizer S, Ostfeld RS, Johnson PT, Kutz S, Harvell CD (2013). Climate change and infectious diseases: from evidence to a predictive framework. Science.

[bib4] Alto BW, Juliano SA (2001). Precipitation and temperature effects on populations of *aedes albopictus* (Diptera: culicidae): implications for range expansion. Journal of Medical Entomology.

[bib5] Amarasekare P, Savage V (2012). A framework for elucidating the temperature dependence of fitness. The American Naturalist.

[bib6] Andriamifidy RF, Tjaden NB, Beierkuhnlein C, Thomas SM (2019). Do we know how mosquito disease vectors will respond to climate change?. Emerging Topics in Life Sciences.

[bib7] Angilletta MJ, Wilson RS, Navas CA, James RS (2003). Tradeoffs and the evolution of thermal reaction norms. Trends in Ecology & Evolution.

[bib8] Angilletta MJ (2009). Thermal Adaptation: A Theoretical and Empirical Synthesis.

[bib9] Angilletta MJ, Huey RB, Frazier MR (2010). Thermodynamic effects on organismal performance: is hotter better?. Physiological and Biochemical Zoology.

[bib10] Armbruster P, Bradshaw WE, Steiner AL, Holzapfel CM (1999). Evolutionary responses to environmental stress by the pitcher-plant mosquito, *Wyeomyia smithii*. Heredity.

[bib11] Ashander J, Chevin L-M, Baskett ML (2016). Predicting evolutionary rescue via evolving plasticity in stochastic environments. Proceedings of the Royal Society B: Biological Sciences.

[bib12] Ashburner M (1989). Drosophila A Laboratory Handbook.

[bib13] Bai CM, Ma G, Cai WZ, Ma CS (2019). Independent and combined effects of daytime heat stress and night-time recovery determine thermal performance. Biology Open.

[bib14] Balanyá J, Oller JM, Huey RB, Gilchrist GW, Serra L (2006). Global genetic change tracks global climate warming in *Drosophila subobscura*. Science.

[bib15] Barbour MA, Greyson-Gaito CJ, Sotoodeh A, Locke B, Bascompte J (2020). Loss of consumers constrains phenotypic evolution in the resulting food web. Evolution Letters.

[bib16] Barghi N, Tobler R, Nolte V, Jakšić AM, Mallard F, Otte KA, Dolezal M, Taus T, Kofler R, Schlötterer C (2019). Genetic redundancy fuels polygenic adaptation in *Drosophila*. PLOS Biology.

[bib17] Barrera R, Amador M, Diaz A, Smith J, Munoz-Jordan JL, Rosario Y (2008). Unusual productivity of *Aedes aegypti* in septic tanks and its implications for dengue control. Medical and Veterinary Entomology.

[bib18] Baskett ML, Gomulkiewicz R (2011). Introgressive hybridization as a mechanism for species rescue. Theoretical Ecology.

[bib19] Bay RA, Rose N, Barrett R, Bernatchez L, Ghalambor CK, Lasky JR, Brem RB, Palumbi SR, Ralph P (2017). Predicting responses to contemporary environmental change using evolutionary response architectures. The American Naturalist.

[bib20] Bedhomme S, Agnew P, Sidobre C, Michalakis Y (2003). Sex-specific reaction norms to intraspecific larval competition in the mosquito *aedes aegypti*. Journal of Evolutionary Biology.

[bib21] Bell G (2013). Evolutionary rescue and the limits of adaptation. Philosophical Transactions of the Royal Society B: Biological Sciences.

[bib22] Bell G (2017). Evolutionary rescue. Annual Review of Ecology, Evolution, and Systematics.

[bib23] Bell G, Gonzalez A (2009). Evolutionary rescue can prevent extinction following environmental change. Ecology Letters.

[bib24] Benedict MQ, Cockburn AF, Seawright JA (1991). Heat-shock mortality and induced thermotolerance in larvae of the mosquito *anopheles albimanus*. Journal of the American Mosquito Control Association.

[bib25] Berend K, Haynes K, MacKenzie CM (2019). Common garden experiments as a dynamic tool for ecological studies of alpine plants and communities in northeastern north america. Rhodora.

[bib26] Berrigan D, Hoffmann AA (1998). Correlations between measures of heat resistance and acclimation in two species of *Drosophila* and their hybrids. Biological Journal of the Linnean Society.

[bib27] Blanford S, Read AF, Thomas MB (2009). Thermal behaviour of *anopheles stephensi* in response to infection with malaria and fungal entomopathogens. Malaria Journal.

[bib28] Blanford JI, Blanford S, Crane RG, Mann ME, Paaijmans KP, Schreiber KV, Thomas MB (2013). Implications of temperature variation for malaria parasite development across Africa. Scientific Reports.

[bib29] Bødker R, Akida J, Shayo D, Kisinza W, Msangeni HA, Pedersen EM, Lindsay SW (2003). Relationship between altitude and intensity of malaria transmission in the Usambara Mountains, Tanzania. Journal of Medical Entomology.

[bib30] Bourne EC, Bocedi G, Travis JMJ, Pakeman RJ, Brooker RW, Schiffers K (2014). Between migration load and evolutionary rescue: dispersal, adaptation and the response of spatially structured populations to environmental change. Proceedings of the Royal Society B: Biological Sciences.

[bib31] Bozinovic F, Bastías DA, Boher F, Clavijo-Baquet S, Estay SA, Angilletta MJ (2011). The mean and variance of environmental temperature interact to determine physiological tolerance and fitness. Physiological and Biochemical Zoology.

[bib32] Bradshaw WE, Fujiyama S, Holzapfel CM (2000). Adaptation to the thermal climate of North America by the pitcher-plant mosquito, Wyeomyia smithii. Ecology.

[bib33] Brady OJ, Johansson MA, Guerra CA, Bhatt S, Golding N, Pigott DM, Delatte H, Grech MG, Leisnham PT, Maciel-de-Freitas R, Styer LM, Smith DL, Scott TW, Gething PW, Hay SI (2013). Modelling adult *Aedesaegypti* and *Aedes* albopictus survival at different temperatures in laboratory and field settings. Parasites & Vectors.

[bib34] Bridle JR, Polechová J, Kawata M, Butlin RK (2010). Why is adaptation prevented at ecological margins? new insights from individual-based simulations. Ecology Letters.

[bib35] Buckley LB, Huey RB (2016). How extreme temperatures impact organisms and the evolution of their thermal tolerance. Integrative and Comparative Biology.

[bib36] Bürger R, Lynch M (1995). Evolution and extinction in A changing environment: a quantitative-genetic analysis. Evolution.

[bib37] Bush A, Mokany K, Catullo R, Hoffmann A, Kellermann V, Sgrò C, McEvey S, Ferrier S (2016). Incorporating evolutionary adaptation in species distribution modelling reduces projected vulnerability to climate change. Ecology Letters.

[bib38] Capblancq T, Fitzpatrick MC, Bay RA, Exposito-Alonso M, Keller SR (2020). Genomic prediction of (Mal)Adaptation across current and future climatic landscapes. Annual Review of Ecology, Evolution, and Systematics.

[bib39] Carlson SM, Cunningham CJ, Westley PA (2014). Evolutionary rescue in a changing world. Trends in Ecology & Evolution.

[bib40] Carrasco D, Lefèvre T, Moiroux N, Pennetier C, Chandre F, Cohuet A (2019). Behavioural adaptations of mosquito vectors to insecticide control. Current Opinion in Insect Science.

[bib41] Castañeda LE, Balanyà J, Rezende EL, Santos M (2013). Vanishing chromosomal inversion clines in *Drosophila subobscura* from Chile: is behavioral thermoregulation to blame?. The American Naturalist.

[bib42] Castañeda LE, Romero-Soriano V, Mesas A, Roff DA, Santos M (2019). Evolutionary potential of thermal preference and heat tolerance in *Drosophila subobscura*. Journal of Evolutionary Biology.

[bib43] Cator LJ, Johnson LR, Mordecai EA, Moustaid FE, Smallwood TRC, LaDeau SL, Johansson MA, Hudson PJ, Boots M, Thomas MB, Power AG, Pawar S (2020). The role of vector trait variation in Vector-Borne disease dynamics. Frontiers in Ecology and Evolution.

[bib44] Catullo RA, Ferrier S, Hoffmann AA (2015). Extending spatial modelling of climate change responses beyond the realized niche: estimating, and accommodating, physiological limits and adaptive evolution. Global Ecology and Biogeography.

[bib45] Chadee DD, Martinez R (2016). *Aedes aegypti* (L.) in Latin american and Caribbean Region: with growing evidence for vector adaptation to climate change?. Acta Tropica.

[bib46] Chen XG, Jiang X, Gu J, Xu M, Wu Y, Deng Y, Zhang C, Bonizzoni M, Dermauw W, Vontas J, Armbruster P, Huang X, Yang Y, Zhang H, He W, Peng H, Liu Y, Wu K, Chen J, Lirakis M, Topalis P, Van Leeuwen T, Hall AB, Jiang X, Thorpe C, Mueller RL, Sun C, Waterhouse RM, Yan G, Tu ZJ, Fang X, James AA (2015). Genome sequence of the asian tiger mosquito, *Aedes albopictus*, reveals insights into its biology, genetics, and evolution. PNAS.

[bib47] Chevin LM, Lande R, Mace GM (2010). Adaptation, plasticity, and extinction in a changing environment: towards a predictive theory. PLOS Biology.

[bib48] Chevin L-M, Collins S, Lefèvre F (2013). Phenotypic plasticity and evolutionary demographic responses to climate change: taking theory out to the field. Functional Ecology.

[bib49] Chevin L-M, Visser ME, Tufto J (2015). Estimating the variation, Autocorrelation, and environmental sensitivity of phenotypic selection. Evolution.

[bib50] Chiang HC, Hodson AC (1950). An analytical study of population growth in *Drosophila melanogaster*. Ecological Monographs.

[bib51] Chouin-Carneiro T, Barreto dos Santos F (2017). Transmission of Major Arboviruses in Brazil: The Role of Aedes Aegypti and Aedes Albopictus Vectors. Page Biological Control of Pest and Vector Insects.

[bib52] Chown SL, Jumbam KR, Sørensen JG, Terblanche JS (2009). Phenotypic variance, plasticity and heritability estimates of critical thermal limits depend on methodological context. Functional Ecology.

[bib53] Christophers SR (1960). Aëdes Aegypti (L.), the Yellow Fever Mosquito: Its Life History, Bionomics, and Structure.

[bib54] Chu VM, Sallum MAM, Moore TE, Lainhart W, Schlichting CD, Conn JE (2019). Regional variation in life history traits and plastic responses to temperature of the major malaria vector *nyssorhynchus darlingi* in Brazil. Scientific Reports.

[bib55] Clausen J, Keck DD, Hiesey WM (1941). Regional differentiation in plant species. The American Naturalist.

[bib56] Collins M, Knutti R, Arblaster J, Dufresne J-L, Fichefet T, Gao X, Jr, T WJG, Johns G, Krinner M, Shongwe A, Weaver J, Wehner M (2013). Long-Term Climate Change: Projections, Commitments and Irreversibility.

[bib57] Coluzzi M, Sabatini A, Petrarca V, Di Deco MA (1979). Chromosomal differentiation and adaptation to human environments in the *Anopheles gambiae* complex. Transactions of the Royal Society of Tropical Medicine and Hygiene.

[bib58] Cotto O, Wessely J, Georges D, Klonner G, Schmid M, Dullinger S, Thuiller W, Guillaume F (2017). A dynamic eco-evolutionary model predicts slow response of alpine plants to climate warming. Nature Communications.

[bib59] Couper LI (2021). Software Heritage.

[bib60] Crow JF, Chung YJ (1967). Measurement of effective generation length in *Drosophila* population cages. Genetics.

[bib61] Culler LE, Ayres MP, Virginia RA (2015). In a warmer arctic, mosquitoes avoid increased mortality from predators by growing faster. Proceedings of the Royal Society B: Biological Sciences.

[bib62] Dao A, Yaro AS, Diallo M, Timbiné S, Huestis DL, Kassogué Y, Traoré AI, Sanogo ZL, Samaké D, Lehmann T (2014). Signatures of aestivation and migration in sahelian malaria mosquito populations. Nature.

[bib63] De Kort H, Vandepitte K, Bruun HH, Closset-Kopp D, Honnay O, Mergeay J (2014). Landscape genomics and a common garden trial reveal adaptive differentiation to temperature across Europe in the tree species *Alnus glutinosa*. Molecular Ecology.

[bib64] de Villemereuil P, Gaggiotti OE, Mouterde M, Till-Bottraud I (2016). Common garden experiments in the genomic era: new perspectives and opportunities. Heredity.

[bib65] Delatte H, Gimonneau G, Triboire A, Fontenille D (2009). Influence of temperature on immature development, survival, longevity, fecundity, and gonotrophic cycles of *aedes albopictus*, vector of Chikungunya and dengue in the indian ocean. Journal of Medical Entomology.

[bib66] Deutsch CA, Tewksbury JJ, Huey RB, Sheldon KS, Ghalambor CK, Haak DC, Martin PR (2008). Impacts of climate warming on terrestrial ectotherms across latitude. PNAS.

[bib67] Dewitt TJ, Sih A, Wilson DS (1998). Costs and limits of phenotypic plasticity. Trends in Ecology & Evolution.

[bib68] Diamond SE (2017). Evolutionary potential of upper thermal tolerance: biogeographic patterns and expectations under climate change: biogeography of evolutionary potential. Annals of the New York Academy of Sciences.

[bib69] Dillon ME, Wang G, Garrity PA, Huey RB (2009). Review: thermal preference in *Drosophila*. Journal of Thermal Biology.

[bib70] Diniz DFA, de Albuquerque CMR, Oliva LO, de Melo-Santos MAV, Ayres CFJ (2017). Diapause and quiescence: dormancy mechanisms that contribute to the geographical expansion of mosquitoes and their evolutionary success. Parasites & Vectors.

[bib71] Diniz‐Filho JAF, Souza KS, Bini LM, Loyola R, Dobrovolski R, Rodrigues JFM, Lima‐Ribeiro S, Terribile LC, Rangel TF, Bione I, Freitas R, Machado IF, Rocha T, Lorini ML, Vale MM, Navas CA, Maciel NM, Villalobos F, Olalla‐Tarraga MA, Gouveia S (2019). A macroecological approach to evolutionary rescue and adaptation to climate change. Ecography.

[bib72] Dodson BL, Kramer LD, Rasgon JL (2012). Effects of larval rearing temperature on immature development and west nile virus vector competence of *Culex tarsalis*. Parasites & Vectors.

[bib73] Dowd WW, King FA, Denny MW (2015). Thermal variation, thermal extremes and the physiological performance of individuals. Journal of Experimental Biology.

[bib74] Egizi A, Fefferman NH, Fonseca DM (2015). Evidence that implicit assumptions of 'no evolution' of disease vectors in changing environments can be violated on a rapid timescale. Philosophical Transactions of the Royal Society B: Biological Sciences.

[bib75] Emiljanowicz LM, Ryan GD, Langille A, Newman J (2014). Development, reproductive output and population growth of the fruit fly pest *Drosophila suzukii* (Diptera: drosophilidae) on artificial diet. Journal of Economic Entomology.

[bib76] Exposito-Alonso M, Vasseur F, Ding W, Wang G, Burbano HA, Weigel D (2018). Genomic basis and evolutionary potential for extreme drought adaptation in *Arabidopsis thaliana*. Nature Ecology & Evolution.

[bib77] Exposito-Alonso M, Burbano HA, Bossdorf O, Nielsen R, Weigel D, 500 Genomes Field Experiment Team (2019). Natural selection on the *Arabidopsis thaliana* genome in present and future climates. Nature.

[bib78] Falconer DS, Mackay T (1996). Introduction to Quantitative Genetics.

[bib79] Fallis LC, Fanara JJ, Morgan TJ (2011). Genetic variation in heat-stress tolerance among south american *Drosophila* populations. Genetica.

[bib80] Faske TM, Thompson LM, Banahene N, Levorse A, Quiroga Herrera M, Sherman K, Timko SE, Yang B, Gray DR, Parry D, Tobin PC, Eckert AJ, Johnson DM, Grayson KL (2019). Can gypsy moth stand the heat? A reciprocal transplant experiment with an invasive forest pest across its southern range margin. Biological Invasions.

[bib81] Feder ME, Blair N, Figueras H (1997). Oviposition site selection: unresponsiveness of *Drosophila* to cues of potential thermal stress. Animal Behaviour.

[bib82] Ferguson HM, Maire N, Takken W, Lyimo IN, Briët O, Lindsay SW, Smith TA (2012). Selection of mosquito life-histories: a hidden weapon against malaria?. Malaria Journal.

[bib83] Fernández-Moreno MA, Farr CL, Kaguni LS, Garesse R (2007). *Drosophila melanogaster* as a model system to study mitochondrial biology. Methods in Molecular Biology.

[bib84] Ferreira CP, Lyra SP, Azevedo F, Greenhalgh D, Massad E (2017). Modelling the impact of the long-term use of insecticide-treated bed nets on *anopheles* mosquito biting time. Malaria Journal.

[bib85] Focks DA, Haile DG, Daniels E, Mount GA (1993). Dynamic life table model for *aedes aegypti* (Diptera: culicidae): analysis of the literature and model development. Journal of Medical Entomology.

[bib86] Fouet C, Gray E, Besansky NJ, Costantini C (2012). Adaptation to aridity in the malaria mosquito *anopheles gambiae*: chromosomal inversion polymorphism and body size influence resistance to desiccation. PLOS ONE.

[bib87] Franklinos LHV, Jones KE, Redding DW, Abubakar I (2019). The effect of global change on mosquito-borne disease. The Lancet Infectious Diseases.

[bib88] Franks SJ, Sim S, Weis AE (2007). Rapid evolution of flowering time by an annual plant in response to a climate fluctuation. PNAS.

[bib89] Friedline CJ, Faske TM, Lind BM, Hobson EM, Parry D, Dyer RJ, Johnson DM, Thompson LM, Grayson KL, Eckert AJ (2019). Evolutionary genomics of gypsy moth populations sampled along a latitudinal gradient. Molecular Ecology.

[bib90] Fuller RC, Baer CF, Travis J (2005). How and when selection experiments might actually be useful. Integrative and Comparative Biology.

[bib91] Garant D, Forde SE, Hendry AP (2007). The multifarious effects of dispersal and gene flow on contemporary adaptation. Functional Ecology.

[bib92] Garland T, Rose MR (2009). Experimental Evolution: Concepts, Methods, and Applications of Selection Experiments.

[bib93] Gething PW, Smith DL, Patil AP, Tatem AJ, Snow RW, Hay SI (2010). Climate change and the global malaria recession. Nature.

[bib94] Gibbs AG, Perkins MC, Markow TA (2003). No place to hide: microclimates of sonoran desert *Drosophila*. Journal of Thermal Biology.

[bib95] Gienapp P, Leimu R, Merilä J (2007). Responses to climate change in avian migration time—microevolution versus phenotypic plasticity. Climate Research.

[bib96] Gienapp P, Teplitsky C, Alho JS, Mills JA, Merilä J (2008). Climate change and evolution: disentangling environmental and genetic responses. Molecular Ecology.

[bib97] Gienapp P, Lof M, Reed TE, McNamara J, Verhulst S, Visser ME (2013). Predicting demographically sustainable rates of adaptation: can great tit breeding time keep pace with climate change?. Philosophical Transactions of the Royal Society B: Biological Sciences.

[bib98] Githeko AK, Service MW, Mbogo CM, Atieli FK (1996). Resting behaviour, ecology and genetics of malaria vectors in large scale agricultural Areas of western Kenya. Parassitologia.

[bib99] Gomulkiewicz R, Houle D (2009). Demographic and genetic constraints on evolution. The American Naturalist.

[bib100] Gomulkiewicz R, Shaw RG (2013). Evolutionary rescue beyond the models. Philosophical Transactions of the Royal Society B: Biological Sciences.

[bib101] Gonzalez A, Ronce O, Ferriere R, Hochberg ME (2013). Evolutionary rescue: an emerging focus at the intersection between ecology and evolution. Philosophical Transactions of the Royal Society B: Biological Sciences.

[bib102] González-Tokman D, Córdoba-Aguilar A, Dáttilo W, Lira-Noriega A, Sánchez-Guillén RA, Villalobos F (2020). Insect responses to heat: physiological mechanisms, evolution and ecological implications in a warming world. Biological Reviews.

[bib103] Gray EM (2013). Thermal acclimation in a complex life cycle: the effects of larval and adult thermal conditions on metabolic rate and heat resistance in *culex pipiens* (Diptera: culicidae). Journal of Insect Physiology.

[bib104] Gunderson AR, Stillman JH (2015). Plasticity in thermal tolerance has limited potential to buffer ectotherms from global warming. Proceedings of the Royal Society B: Biological Sciences.

[bib105] Haller BC, Messer PW (2017). SLiM 2: flexible, interactive forward genetic simulations. Molecular Biology and Evolution.

[bib106] Hangartner S, Hoffmann AA (2016). Evolutionary potential of multiple measures of upper thermal tolerance in *Drosophila melanogaster*. Functional Ecology.

[bib107] Hartl DL, Clark AG (1997). Principles of Population Genetics.

[bib108] Harvell CD, Mitchell CE, Ward JR, Altizer S, Dobson AP, Ostfeld RS, Samuel MD (2002). Climate warming and disease risks for terrestrial and marine biota. Science.

[bib109] Haufe WO, Burgess L (1956). Development of *Aedes* (Diptera: Culicidae) at Fort Churchill, Manitoba, and Prediction of Dates of Emergence. Ecology.

[bib110] Heerwaarden B, Kellermann V, Sgrò CM (2016). Limited scope for plasticity to increase upper thermal limits. Functional Ecology.

[bib111] Hoffmann AA, Chown SL, Clusella-Trullas S (2013). Upper thermal limits in terrestrial ectotherms: how constrained are they?. Functional Ecology.

[bib112] Hoffmann AA, Sgrò CM (2011). Climate change and evolutionary adaptation. Nature.

[bib113] Hoffmann AA, Watson M (1993). Geographical variation in the acclimation responses of *Drosophila* to temperature extremes. The American Naturalist.

[bib114] Holt RA, Subramanian GM, Halpern A, Sutton GG, Charlab R, Nusskern DR, Wincker P, Clark AG, Ribeiro JM, Wides R, Salzberg SL, Loftus B, Yandell M, Majoros WH, Rusch DB, Lai Z, Kraft CL, Abril JF, Anthouard V, Arensburger P, Atkinson PW, Baden H, de Berardinis V, Baldwin D, Benes V, Biedler J, Blass C, Bolanos R, Boscus D, Barnstead M, Cai S, Center A, Chaturverdi K, Christophides GK, Chrystal MA, Clamp M, Cravchik A, Curwen V, Dana A, Delcher A, Dew I, Evans CA, Flanigan M, Grundschober-Freimoser A, Friedli L, Gu Z, Guan P, Guigo R, Hillenmeyer ME, Hladun SL, Hogan JR, Hong YS, Hoover J, Jaillon O, Ke Z, Kodira C, Kokoza E, Koutsos A, Letunic I, Levitsky A, Liang Y, Lin JJ, Lobo NF, Lopez JR, Malek JA, McIntosh TC, Meister S, Miller J, Mobarry C, Mongin E, Murphy SD, O'Brochta DA, Pfannkoch C, Qi R, Regier MA, Remington K, Shao H, Sharakhova MV, Sitter CD, Shetty J, Smith TJ, Strong R, Sun J, Thomasova D, Ton LQ, Topalis P, Tu Z, Unger MF, Walenz B, Wang A, Wang J, Wang M, Wang X, Woodford KJ, Wortman JR, Wu M, Yao A, Zdobnov EM, Zhang H, Zhao Q, Zhao S, Zhu SC, Zhimulev I, Coluzzi M, della Torre A, Roth CW, Louis C, Kalush F, Mural RJ, Myers EW, Adams MD, Smith HO, Broder S, Gardner MJ, Fraser CM, Birney E, Bork P, Brey PT, Venter JC, Weissenbach J, Kafatos FC, Collins FH, Hoffman SL (2002). The genome sequence of the malaria mosquito *anopheles gambiae*. Science.

[bib115] Huey RB, Partridge L, Fowler K (1991). Thermal sensitivity of *Drosophila melanogaster* responds rapidly to laboratory natural selection. Evolution.

[bib116] Huey RB, Crill WD, Kingsolver JG, Weber KE (1992). A method for rapid measurement of heat or cold resistance of small insects. Functional Ecology.

[bib117] Huey RB, Berrigan D (2001). Temperature, demography, and ectotherm fitness. The American Naturalist.

[bib118] Huey RB, Kingsolver JG (1993). Evolution of resistance to high temperature in ectotherms. The American Naturalist.

[bib119] Huey RB, Kingsolver JG (2019). Climate warming, resource availability, and the metabolic meltdown of ectotherms. The American Naturalist.

[bib120] Huey RB, Pascual M (2009). Partial thermoregulatory compensation by a rapidly evolving invasive species along a latitudinal cline. Ecology.

[bib121] Huey RB, Stevenson RD (1979). Integrating thermal physiology and ecology of ectotherms: a discussion of approaches. American Zoologist.

[bib122] população E, Instituto Brasileiro de Geografia e Estatistica (2016). IBGE. https://www.ibge.gov.br.

[bib123] IPCC (2007). Climate Change 2007: The Physical Science Basis.

[bib124] Jenkins NL, Hoffmann AA (1994). Genetic and maternal variation for heat resistance in *Drosophila* from the field. Genetics.

[bib125] Jiang X, Peery A, Hall AB, Sharma A, Chen XG, Waterhouse RM, Komissarov A, Riehle MM, Shouche Y, Sharakhova MV, Lawson D, Pakpour N, Arensburger P, Davidson VL, Eiglmeier K, Emrich S, George P, Kennedy RC, Mane SP, Maslen G, Oringanje C, Qi Y, Settlage R, Tojo M, Tubio JM, Unger MF, Wang B, Vernick KD, Ribeiro JM, James AA, Michel K, Riehle MA, Luckhart S, Sharakhov IV, Tu Z (2014). Genome analysis of a major urban malaria vector mosquito, anopheles stephensi. Genome Biology.

[bib126] Johnson LR, Ben-Horin T, Lafferty KD, McNally A, Mordecai E, Paaijmans KP, Pawar S, Ryan SJ (2015). Understanding uncertainty in temperature effects on vector-borne disease: a bayesian approach. Ecology.

[bib127] Johnson EE, Escobar LE, Zambrana-Torrelio C (2019). An ecological framework for modeling the geography of disease transmission. Trends in Ecology & Evolution.

[bib128] Joubert DA, Walker T, Carrington LB, De Bruyne JT, Kien DH, Hoang NT, Chau NV, Iturbe-Ormaetxe I, Simmons CP, O'Neill SL (2016). Establishment of a *Wolbachia* superinfection in *Aedes aegypti* Mosquitoes as a Potential Approach for Future Resistance Management. PLOS Pathogens.

[bib129] Kamimura K, Matsuse IT, Takahashi H, Komukai J, Fukuda T, Suzuki K, Aratani M, Shirai Y, Mogi M (2002). Effect of temperature on the development of *aedes* aegypti and *Aedes* albopictus. Medical Entomology and Zoology.

[bib130] Karell P, Ahola K, Karstinen T, Valkama J, Brommer JE (2011). Climate change drives microevolution in a wild bird. Nature Communications.

[bib131] Kauffman E, Payne A, Franke M, Schmid M, Harris E, Kramer L (2017). Rearing of *culex spp.* and *aedes spp*. mosquitoes. Bio-Protocol.

[bib132] Kaufman MG, Fonseca DM (2014). Invasion biology of *Aedes japonicus japonicus* (Diptera: culicidae). Annual Review of Entomology.

[bib133] Kearney M, Porter WP, Williams C, Ritchie S, Hoffmann AA (2009). Integrating biophysical models and evolutionary theory to predict climatic impacts on species’ ranges: the dengue mosquito *Aedes aegypti* in Australia. Functional Ecology.

[bib134] Kellermann V, Overgaard J, Hoffmann AA, Fløjgaard C, Svenning JC, Loeschcke V (2012). Upper thermal limits of *Drosophila* are linked to species distributions and strongly constrained phylogenetically. PNAS.

[bib135] Kingsolver JG (2009). The well-temperatured biologist. (American society of naturalists presidential address). The American Naturalist.

[bib136] Kingsolver JG, Diamond SE, Buckley LB (2013). Heat stress and the fitness consequences of climate change for terrestrial ectotherms. Functional Ecology.

[bib137] Kirkpatrick M, Peischl S (2013). Evolutionary rescue by beneficial mutations in environments that change in space and time. Philosophical Transactions of the Royal Society B: Biological Sciences.

[bib138] Koella JC, Boëte C (2002). A genetic correlation between age at Pupation and melanization immune response of the yellow fever mosquito *Aedes aegypti*. Evolution.

[bib139] Kontopoulos DG, Sebille E, Lange M, Yvon‐Durocher G, Barraclough TG, Pawar S (2020). Phytoplankton thermal responses adapt in the absence of hard thermodynamic constraints. Evolution.

[bib140] Kopp M, Matuszewski S (2014). Rapid evolution of quantitative traits: theoretical perspectives. Evolutionary Applications.

[bib141] Kovach RP, Gharrett AJ, Tallmon DA (2012). Genetic change for earlier migration timing in a pink salmon population. Proceedings of the Royal Society B: Biological Sciences.

[bib142] Kristensen TN, Kjeldal H, Schou MF, Nielsen JL (2016). Proteomic data reveals a physiological basis for costs and benefits associated with thermal acclimation. Journal of Experimental Biology.

[bib143] Lafferty KD, Mordecai EA (2016). The rise and fall of infectious disease in a warmer world. F1000Research.

[bib144] Lambrechts L, Paaijmans KP, Fansiri T, Carrington LB, Kramer LD, Thomas MB, Scott TW (2011). Impact of daily temperature fluctuations on dengue virus transmission by aedes aegypti. PNAS.

[bib145] Lande R (1976). Natural selection and random genetic drift in phenotypic evolution. Evolution.

[bib146] Lande R, Arnold SJ (1983). The measurement of selection on correlated characters. Evolution.

[bib147] Larish LB, Savage HM (2005). Introduction and establishment of Aedes (Finlaya) *Japonicus japonicus* (Theobald) on the island of hawaii: implications for arbovirus transmission. Journal of the American Mosquito Control Association.

[bib148] Latimer CA, Wilson RS, Chenoweth SF (2011). Quantitative genetic variation for thermal performance curves within and among natural populations of *Drosophila serrata*. Journal of Evolutionary Biology.

[bib149] Le Goff G, Damiens D, Ruttee AH, Payet L, Lebon C, Dehecq JS, Gouagna LC (2019). Field evaluation of seasonal trends in relative population sizes and dispersal pattern of *aedes albopictus* males in support of the design of a sterile male release strategy. Parasites & Vectors.

[bib150] Lehmann T, Hawley WA, Grebert H, Collins FH (1998). The effective population size of *anopheles gambiae* in Kenya: implications for population structure. Molecular Biology and Evolution.

[bib151] Lehmann T, Dao A, Yaro AS, Adamou A, Kassogue Y, Diallo M, Sékou T, Coscaron-Arias C (2010). Aestivation of the african malaria mosquito, *anopheles gambiae* in the sahel. The American Journal of Tropical Medicine and Hygiene.

[bib152] Lehmann T, Dao A, Yaro AS, Diallo M, Timbiné S, Huestis DL, Adamou A, Kassogué Y, Traoré AI (2014). Seasonal variation in spatial distributions of *anopheles gambiae* in a sahelian village: evidence for aestivation. Journal of Medical Entomology.

[bib153] Lehmann P, Ammunét T, Barton M, Battisti A, Eigenbrode SD, Jepsen JU, Kalinkat G, Neuvonen S, Niemelä P, Terblanche JS, Økland B, Björkman C (2020). Complex responses of global insect pests to climate warming. Frontiers in Ecology and the Environment.

[bib154] Lin Q-C, Zhai Y-F, Zhang A-S, Men X-Y, Zhang X-Y, Zalom FG, Zhou C-G, Yu Y (2014). Comparative Developmental Times and Laboratory Life Tables for *Drosophlia suzukii* and *Drosophila melanogaster* (Diptera: Drosophilidae). Florida Entomologist.

[bib155] Lockwood BL, Gupta T, Scavotto R (2018). Disparate patterns of thermal adaptation between life stages in temperate vs. tropical *Drosophila melanogaster*. Journal of Evolutionary Biology.

[bib156] Loeschcke V, Hoffmann AA (2007). Consequences of heat hardening on a field fitness component in *Drosophila* depend on environmental temperature. The American Naturalist.

[bib157] Logan ML, Curlis JD, Gilbert AL, Miles DB, Chung AK, McGlothlin JW, Cox RM (2018). Thermal physiology and thermoregulatory behaviour exhibit low heritability despite genetic divergence between lizard populations. Proceedings of the Royal Society B: Biological Sciences.

[bib158] Lynch M, Lande R (1993). Evolution and extinction in response to environmental change. Biotic Interactions and Global Change.

[bib159] Lyons CL, Coetzee M, Terblanche JS, Chown SL (2012). Thermal limits of wild and laboratory strains of two african malaria vector species, *Anopheles arabiensis* and *Anopheles funestus*. Malaria Journal.

[bib160] Ma CS, Ma G, Pincebourde S (2021). Survive a warming climate: insect responses to extreme high temperatures. Annual Review of Entomology.

[bib161] Macdonald G (1952). The analysis of the sporozoite rate. Tropical Diseases Bulletin.

[bib162] Maciel-de-Freitas R, Eiras ÁE, Lourenço-de-Oliveira R (2008). Calculating the survival rate and estimated population density of gravid *Aedes aegypti* (Diptera, Culicidae) in Rio de Janeiro, Brazil. Cadernos De Saúde Pública.

[bib163] MacLean HJ, Sørensen JG, Kristensen TN, Loeschcke V, Beedholm K, Kellermann V, Overgaard J (2019). Evolution and plasticity of thermal performance: an analysis of variation in thermal tolerance and fitness in 22 *Drosophila* species. Philosophical Transactions of the Royal Society B: Biological Sciences.

[bib164] Manda H, Arce LM, Foggie T, Shah P, Grieco JP, Achee NL (2011). Effects of irritant chemicals on aedes aegypti resting behavior: is there a simple shift to untreated "safe sites"?. PLOS Neglected Tropical Diseases.

[bib165] Manenti T, Sørensen JG, Moghadam NN, Loeschcke V (2014). Predictability rather than amplitude of temperature fluctuations determines stress resistance in a natural population of *Drosophila simulans*. Journal of Evolutionary Biology.

[bib166] Marinotti O, Cerqueira GC, de Almeida LG, Ferro MI, Loreto EL, Zaha A, Teixeira SM, Wespiser AR, Almeida E Silva A, Schlindwein AD, Pacheco AC, Silva AL, Graveley BR, Walenz BP, Lima BA, Ribeiro CA, Nunes-Silva CG, de Carvalho CR, Soares CM, de Menezes CB, Matiolli C, Caffrey D, Araújo DA, de Oliveira DM, Golenbock D, Grisard EC, Fantinatti-Garboggini F, de Carvalho FM, Barcellos FG, Prosdocimi F, May G, Azevedo Junior GM, Guimarães GM, Goldman GH, Padilha IQ, Batista JS, Ferro JA, Ribeiro JM, Fietto JL, Dabbas KM, Cerdeira L, Agnez-Lima LF, Brocchi M, de Carvalho MO, Teixeira MM, Diniz Maia MM, Goldman MH, Cruz Schneider MP, Felipe MS, Hungria M, Nicolás MF, Pereira M, Montes MA, Cantão ME, Vincentz M, Rafael MS, Silverman N, Stoco PH, Souza RC, Vicentini R, Gazzinelli RT, Neves RO, Silva R, Astolfi-Filho S, Maciel TE, Urményi TP, Tadei WP, Camargo EP, de Vasconcelos AT (2013). The genome of *anopheles darlingi*, the main neotropical malaria vector. Nucleic Acids Research.

[bib167] Martin G, Aguilée R, Ramsayer J, Kaltz O, Ronce O (2013). The probability of evolutionary rescue: towards a quantitative comparison between theory and evolution experiments. Philosophical Transactions of the Royal Society B: Biological Sciences.

[bib168] Matsumura S, Arlinghaus R, Dieckmann U (2012). Standardizing selection strengths to study selection in the wild: a critical comparison and suggestions for the future. BioScience.

[bib169] Matthews BJ, Dudchenko O, Kingan SB, Koren S, Antoshechkin I, Crawford JE, Glassford WJ, Herre M, Redmond SN, Rose NH, Weedall GD, Wu Y, Batra SS, Brito-Sierra CA, Buckingham SD, Campbell CL, Chan S, Cox E, Evans BR, Fansiri T, Filipović I, Fontaine A, Gloria-Soria A, Hall R, Joardar VS, Jones AK, Kay RGG, Kodali VK, Lee J, Lycett GJ, Mitchell SN, Muehling J, Murphy MR, Omer AD, Partridge FA, Peluso P, Aiden AP, Ramasamy V, Rašić G, Roy S, Saavedra-Rodriguez K, Sharan S, Sharma A, Smith ML, Turner J, Weakley AM, Zhao Z, Akbari OS, Black WC, Cao H, Darby AC, Hill CA, Johnston JS, Murphy TD, Raikhel AS, Sattelle DB, Sharakhov IV, White BJ, Zhao L, Aiden EL, Mann RS, Lambrechts L, Powell JR, Sharakhova MV, Tu Z, Robertson HM, McBride CS, Hastie AR, Korlach J, Neafsey DE, Phillippy AM, Vosshall LB (2018). Improved reference genome of *aedes aegypti* informs arbovirus vector control. Nature.

[bib170] Matz MV, Treml EA, Haller BC (2020). Estimating the potential for coral adaptation to global warming across the Indo-West pacific. Global Change Biology.

[bib171] McColl G, Hoffmann AA, McKechnie SW (1996). Response of two heat shock genes to selection for knockdown heat resistance in *Drosophila melanogaster*. Genetics.

[bib172] Medley KA (2010). Niche shifts during the global invasion of the Asian tiger mosquito, *Aedes albopictus* Skuse (Culicidae), revealed by reciprocal distribution models. Global Ecology and Biogeography.

[bib173] Medley KA, Westby KM, Jenkins DG (2019). Rapid local adaptation to northern winters in the invasive Asian tiger mosquito *Aedes albopictus* : A moving target. Journal of Applied Ecology.

[bib174] Merilä J, Hendry AP (2014). Climate change, adaptation, and phenotypic plasticity: the problem and the evidence. Evolutionary Applications.

[bib175] Minakawa N, Yan G, Githeko A, Zhou G, Omukunda E (2006). Malaria vector productivity in relation to the Highland environment in Kenya. The American Journal of Tropical Medicine and Hygiene.

[bib176] Mitchell KA, Hoffmann AA (2010). Thermal ramping rate influences evolutionary potential and species differences for upper thermal limits in *Drosophila*. Functional Ecology.

[bib177] Mogi M (1992). Temperature and photoperiod effects on larval and ovarian development of New Zealand strains of *Culex quinquefasciatus* (Diptera: Culicidae). Annals of the Entomological Society of America.

[bib178] Moiroux N, Gomez MB, Pennetier C, Elanga E, Djènontin A, Chandre F, Djègbé I, Guis H, Corbel V (2012). Changes in anopheles funestus biting behavior following universal coverage of long-lasting insecticidal nets in Benin. The Journal of Infectious Diseases.

[bib179] Montgomery ME, Wallner WE, Berryman A. A (1988). The Gypsy Moth. Dynamics of Forest Insect Populations: Patterns, Causes, Implications.

[bib180] Moran EV, Alexander JM (2014). Evolutionary responses to global change: lessons from invasive species. Ecology Letters.

[bib181] Mordecai EA, Paaijmans KP, Johnson LR, Balzer C, Ben-Horin T, de Moor E, McNally A, Pawar S, Ryan SJ, Smith TC, Lafferty KD (2013). Optimal temperature for malaria transmission is dramatically lower than previously predicted. Ecology Letters.

[bib182] Mordecai EA, Cohen JM, Evans MV, Gudapati P, Johnson LR, Lippi CA, Miazgowicz K, Murdock CC, Rohr JR, Ryan SJ, Savage V, Shocket MS, Stewart Ibarra A, Thomas MB, Weikel DP (2017). Detecting the impact of temperature on transmission of zika, dengue, and Chikungunya using mechanistic models. PLOS Neglected Tropical Diseases.

[bib183] Mordecai EA, Caldwell JM, Grossman MK, Lippi CA, Johnson LR, Neira M, Rohr JR, Ryan SJ, Savage V, Shocket MS, Sippy R, Stewart Ibarra AM, Thomas MB, Villena O (2019). Thermal biology of mosquito-borne disease. Ecology Letters.

[bib184] Mordecai EA, Ryan SJ, Caldwell JM, Shah MM, LaBeaud AD (2020). Climate change could shift disease burden from malaria to arboviruses in africa. The Lancet Planetary Health.

[bib185] Mousseau TA, Roff DA (1987). Natural selection and the heritability of fitness components. Heredity.

[bib186] Mueller LD, Ayala FJ (1981). Trade-off between r-selection and K-selection in *Drosophila* populations. PNAS.

[bib187] Munstermann LE, Crampton J. M, Beard C. B, Louis C (1997). Care and maintenance of Aedes mosquito colonies. The Molecular Biology of Insect Disease Vectors: A Methods Manual.

[bib188] Murdock CC, Paaijmans KP, Bell AS, King JG, Hillyer JF, Read AF, Thomas MB (2012). Complex effects of temperature on mosquito immune function. Proceedings of the Royal Society B: Biological Sciences.

[bib189] Muturi EJ, Lampman R, Costanzo K, Alto BW (2011). Effect of temperature and insecticide stress on life-history traits of *culex* restuans and *aedes albopictus* (Diptera: culicidae). Journal of Medical Entomology.

[bib190] National Notifiable Diseases Information System (SINAN) (2019). DATASUS – Ministério da Saúde. https://datasus.saude.gov.br/.

[bib191] Nayar JK, Sauerman DM (1971). The effects of diet on life-span, fecundity and flight potential of *aedes taeniorhynchus* adults. Journal of Medical Entomology.

[bib192] Neira M, Lacroix R, Cáceres L, Kaiser PE, Young J, Pineda L, Black I, Sosa N, Nimmo D, Alphey L, McKemey A (2014). Estimation of *aedes aegypti* (Diptera: Culicidae) population size and adult male survival in an urban area in Panama. Memórias Do Instituto Oswaldo Cruz.

[bib193] Nene V, Wortman JR, Lawson D, Haas B, Kodira C, Tu ZJ, Loftus B, Xi Z, Megy K, Grabherr M, Ren Q, Zdobnov EM, Lobo NF, Campbell KS, Brown SE, Bonaldo MF, Zhu J, Sinkins SP, Hogenkamp DG, Amedeo P, Arensburger P, Atkinson PW, Bidwell S, Biedler J, Birney E, Bruggner RV, Costas J, Coy MR, Crabtree J, Crawford M, Debruyn B, Decaprio D, Eiglmeier K, Eisenstadt E, El-Dorry H, Gelbart WM, Gomes SL, Hammond M, Hannick LI, Hogan JR, Holmes MH, Jaffe D, Johnston JS, Kennedy RC, Koo H, Kravitz S, Kriventseva EV, Kulp D, Labutti K, Lee E, Li S, Lovin DD, Mao C, Mauceli E, Menck CF, Miller JR, Montgomery P, Mori A, Nascimento AL, Naveira HF, Nusbaum C, O'leary S, Orvis J, Pertea M, Quesneville H, Reidenbach KR, Rogers YH, Roth CW, Schneider JR, Schatz M, Shumway M, Stanke M, Stinson EO, Tubio JM, Vanzee JP, Verjovski-Almeida S, Werner D, White O, Wyder S, Zeng Q, Zhao Q, Zhao Y, Hill CA, Raikhel AS, Soares MB, Knudson DL, Lee NH, Galagan J, Salzberg SL, Paulsen IT, Dimopoulos G, Collins FH, Birren B, Fraser-Liggett CM, Severson DW (2007). Genome sequence of *aedes aegypti,* a major arbovirus vector. Science.

[bib194] Nordstrom S, Hufbauer RA, Melbourne B (2020). Negative density dependence constrains evolutionary rescue.

[bib195] Nussey DH, Postma E, Gienapp P, Visser ME (2005). Selection on heritable phenotypic plasticity in a wild bird population. Science.

[bib196] Orr HA, Unckless RL (2008). Population extinction and the genetics of adaptation. The American Naturalist.

[bib197] Overgaard J, Kristensen TN, Mitchell KA, Hoffmann AA (2011). Thermal tolerance in widespread and tropical *Drosophila* species: does phenotypic plasticity increase with latitude?. The American Naturalist.

[bib198] Ożgo M, Schilthuizen M (2012). Evolutionary change in *Cepaea nemoralis* shell colour over 43 years. Global Change Biology.

[bib199] Paaijmans KP, Takken W, Githeko AK, Jacobs AF (2008). The effect of water turbidity on the near-surface water temperature of larval habitats of the malaria mosquito anopheles gambiae. International Journal of Biometeorology.

[bib200] Paaijmans KP, Imbahale SS, Thomas MB, Takken W (2010). Relevant microclimate for determining the development rate of malaria mosquitoes and possible implications of climate change. Malaria Journal.

[bib201] Paaijmans KP, Blanford S, Chan BH, Thomas MB (2012). Warmer temperatures reduce the vectorial capacity of malaria mosquitoes. Biology Letters.

[bib202] Paaijmans KP, Heinig RL, Seliga RA, Blanford JI, Blanford S, Murdock CC, Thomas MB (2013). Temperature variation makes ectotherms more sensitive to climate change. Global Change Biology.

[bib203] Paaijmans KP, Thomas MB (2011). The influence of mosquito resting behaviour and associated microclimate for malaria risk. Malaria Journal.

[bib204] Papadopoulos NT, Carey JR, Ioannou CS, Ji H, Müller H-G, Wang J-L, Luckhart S, Lewis EE (2016). Seasonality of Post-capture longevity in a Medically-Important mosquito (*Culex pipiens*). Frontiers in Ecology and Evolution.

[bib205] Pates H, Curtis C (2005). Mosquito behavior and vector control. Annual Review of Entomology.

[bib206] Patz JA, Olson SH, Uejio CK, Gibbs HK (2008). Disease emergence from global climate and land use change. Medical Clinics of North America.

[bib207] Patz JA, Reisen WK (2001). Immunology, climate change and vector-borne diseases. Trends in Immunology.

[bib208] Pinsky ML, Eikeset AM, McCauley DJ, Payne JL, Sunday JM (2019). Greater vulnerability to warming of marine versus terrestrial ectotherms. Nature.

[bib209] Porter JH, Parry ML, Carter TR (1991). The potential effects of climatic change on agricultural insect pests. Agricultural and Forest Meteorology.

[bib210] Rashkovetsky E, Iliadi K, Michalak P, Lupu A, Nevo E, Feder ME, Korol A (2006). Adaptive differentiation of thermotolerance in *Drosophila* along a microclimatic gradient. Heredity.

[bib211] Réale D, McAdam AG, Boutin S, Berteaux D (2003). Genetic and plastic responses of a northern mammal to climate change. Proceedings of the Royal Society of London. Series B: Biological Sciences.

[bib212] Reeves W, Korecki J (2004). Ochlerotatus japonicus japonicus (Theobald) (Diptera: culicidae), a new invasive mosquito for Georgia and south Carolina. Proceedings of the Entomological Society of Washington.

[bib213] Reisen WK (1995). Effect of temperature on *culex tarsalis* (Diptera: culicidae) from the Coachella and san joaquin valleys of California. Journal of Medical Entomology.

[bib214] Reisen WK, Aslamkhan M (1978). Biting rhythms of some Pakistan mosquitoes (Diptera: culicidae). Bulletin of Entomological Research.

[bib215] Reiter P (2001). Climate change and mosquito-borne disease. Environmental Health Perspectives.

[bib216] Rezende EL, Bozinovic F, Szilágyi A, Santos M (2020). Predicting temperature mortality and selection in natural *Drosophila* populations. Science.

[bib217] Riahi K, Rao S, Krey V, Cho C, Chirkov V, Fischer G, Kindermann G, Nakicenovic N, Rafaj P (2011). RCP 8.5—A scenario of comparatively high greenhouse gas emissions. Climatic Change.

[bib218] Rocca KA, Gray EM, Costantini C, Besansky NJ (2009). 2la chromosomal inversion enhances thermal tolerance of *Anopheles gambiae* larvae. Malaria Journal.

[bib219] Rocklöv J, Dubrow R (2020). Climate change: an enduring challenge for vector-borne disease prevention and control. Nature Immunology.

[bib220] Rodríguez-Trelles F, Rodríguez MA (1998). Rapid micro-evolution and loss of chromosomal diversity in *Drosophila* in response to climate warming. Evolutionary Ecology.

[bib221] Roff DA, Mousseau TA (1987). Quantitative genetics and fitness: lessons from *Drosophila*. Heredity.

[bib222] Rohr JR, Dobson AP, Johnson PT, Kilpatrick AM, Paull SH, Raffel TR, Ruiz-Moreno D, Thomas MB (2011). Frontiers in climate change-disease research. Trends in Ecology & Evolution.

[bib223] Rohr JR, Civitello DJ, Cohen JM, Roznik EA, Sinervo B, Dell AI (2018). The complex drivers of thermal acclimation and breadth in ectotherms. Ecology Letters.

[bib224] Rohr JR, Cohen JM (2020). Understanding how temperature shifts could impact infectious disease. PLOS Biology.

[bib225] Rolandi C, Lighton JRB, de la Vega GJ, Schilman PE, Mensch J (2018). Genetic variation for tolerance to high temperatures in a population of *Drosophila melanogaster*. Ecology and Evolution.

[bib226] Román-Palacios C, Wiens JJ (2020). Recent responses to climate change reveal the drivers of species extinction and survival. PNAS.

[bib227] Rudolph KE, Lessler J, Moloney RM, Kmush B, Cummings DA (2014). Incubation periods of mosquito-borne viral infections: a systematic review. The American Journal of Tropical Medicine and Hygiene.

[bib228] Ruybal JE, Kramer LD, Kilpatrick AM (2016). Geographic variation in the response of *culex pipiens* life history traits to temperature. Parasites & Vectors.

[bib229] Ryan SJ, McNally A, Johnson LR, Mordecai EA, Ben-Horin T, Paaijmans K, Lafferty KD (2015). Mapping physiological suitability limits for malaria in africa under climate change. Vector-Borne and Zoonotic Diseases.

[bib230] Ryan SJ, Carlson CJ, Mordecai EA, Johnson LR (2019). Global expansion and redistribution of *Aedes*-borne virus transmission risk with climate change. PLOS Neglected Tropical Diseases.

[bib231] Scheiner SM, Barfield M, Holt RD (2017). The genetics of phenotypic plasticity. XV. Genetic assimilation, the baldwin effect, and evolutionary rescue. Ecology and Evolution.

[bib232] Scheiner SM, Berrigan D (1998). The genetics of phenotypic plasticity. VIII. The cost of plasticity in Daphnia pulex. Evolution.

[bib233] Schiffers K, Bourne EC, Lavergne S, Thuiller W, Travis JM (2013). Limited evolutionary rescue of locally adapted populations facing climate change. Philosophical Transactions of the Royal Society B: Biological Sciences.

[bib234] Sears MW, Angilletta MJ, Schuler MS, Borchert J, Dilliplane KF, Stegman M, Rusch TW, Mitchell WA (2016). Configuration of the thermal landscape determines thermoregulatory performance of ectotherms. PNAS.

[bib235] Sgrò CM, Overgaard J, Kristensen TN, Mitchell KA, Cockerell FE, Hoffmann AA (2010). A comprehensive assessment of geographic variation in heat tolerance and hardening capacity in populations of *Drosophila melanogaster* from eastern Australia. Journal of Evolutionary Biology.

[bib236] Shapiro LLM, Whitehead SA, Thomas MB (2017). Quantifying the effects of temperature on mosquito and parasite traits that determine the transmission potential of human malaria. PLOS Biology.

[bib237] Shocket MS, Ryan SJ, Mordecai EA (2018). Temperature explains broad patterns of ross river virus transmission. eLife.

[bib238] Shocket MS, Verwillow AB, Numazu MG, Slamani H, Cohen JM, El Moustaid F, Rohr J, Johnson LR, Mordecai EA (2020). Transmission of west nile and five other temperate mosquito-borne viruses peaks at temperatures between 23°C and 26°C. eLife.

[bib239] Shocket MS, Anderson CB, Caldwell JM, Childs ML, Couper LI, Han S, Harris MJ, Howard ME, MacDonald AJ, Nova N, Mordecai EA, Shocket M. S (2021). Environmental drivers of vector-borne disease. Population Biology of Vector-Borne Diseases.

[bib240] Shragai T, Tesla B, Murdock C, Harrington LC (2017). Zika and Chikungunya: mosquito-borne viruses in a changing world. Annals of the New York Academy of Sciences.

[bib241] Siddiqui WH, Barlow CA (1972). Population growth of *Drosophila melanogaster* (Diptera: drosophilidae) at constant and alternating Temperatures1. Annals of the Entomological Society of America.

[bib242] Simard F, Ayala D, Kamdem GC, Pombi M, Etouna J, Ose K, Fotsing JM, Fontenille D, Besansky NJ, Costantini C (2009). Ecological niche partitioning between *Anopheles gambiae* molecular forms in Cameroon: the ecological side of speciation. BMC Ecology.

[bib243] Sivan A, Shriram AN, Vanamail P, Sugunan AP (2021). Thermotolerance and acclimation in the immature stages of *aedes aegypti* (L) (Diptera: Culicidae) to simulated thermal stress. International Journal of Tropical Insect Science.

[bib244] Somero GN (1995). Proteins and temperature. Annual Review of Physiology.

[bib245] Somero GN (2003). Protein adaptations to temperature and pressure: complementary roles of adaptive changes in amino acid sequence and internal milieu. Comparative Biochemistry and Physiology Part B: Biochemistry and Molecular Biology.

[bib246] Somero GN (2010). The physiology of climate change: how potentials for acclimatization and genetic adaptation will determine ‘winners’ and ‘losers’. Journal of Experimental Biology.

[bib247] Somero G, Lockwood B, Tomanek L (2016). Response to Environmental Challenges from Life's Origins to the Anthropocene. Biochemical Adaptation.

[bib248] Somers G, Brown JE, Barrera R, Powell JR (2011). Genetics and morphology of *aedes aegypti* (Diptera: culicidae) in septic tanks in puerto rico. Journal of Medical Entomology.

[bib249] Sørensen JG, Dahlgaard J, Loeschcke V (2001). Genetic variation in thermal tolerance among natural populations of *Drosophila buzzatii* : down regulation of Hsp70 expression and variation in heat stress resistance traits. Functional Ecology.

[bib250] Stearns SC, Ackermann M, Doebeli M, Kaiser M (2000). Experimental evolution of aging, growth, and reproduction in fruitflies. PNAS.

[bib251] Stinchcombe JR, Dorn LA, Schmitt J (2004). Flowering time plasticity in *Arabidopsis thaliana*: a reanalysis of Westerman & Lawrence (1970). Journal of Evolutionary Biology.

[bib252] Suzuki R, Xu J, Motoya K (2006). Global analyses of satellite-derived vegetation index related to climatological wetness and warmth. International Journal of Climatology.

[bib253] Swallow J, Hayes J, Koteja P, Garland T, Garland T, Rose M. R (2009). Selection Experiments and Experimental Evolution of Performance and Physiology. Experimental Evolution: Concepts, Methods, and Applications of Selection Experiments.

[bib254] Takken W (2002). Do insecticide-treated bednets have an effect on malaria vectors?. Tropical Medicine and International Health.

[bib255] Tatar M, Chien SA, Priest NK (2001). Negligible senescence during reproductive dormancy in *Drosophila melanogaster*. The American Naturalist.

[bib256] Taylor B (1975). Changes in the feeding behaviour of a malaria vector, *anopheles farauti* Lav., following use of DDT as a residual spray in houses in the British Solomon Islands Protectorate. Transactions of the Royal Entomological Society of London.

[bib257] Terblanche JS, Deere JA, Clusella-Trullas S, Janion C, Chown SL (2007). Critical thermal limits depend on methodological context. Proceedings of the Royal Society B: Biological Sciences.

[bib258] Tesla B, Demakovsky LR, Mordecai EA, Ryan SJ, Bonds MH, Ngonghala CN, Brindley MA, Murdock CC (2018). Temperature drives Zika virus transmission: evidence from empirical and mathematical models. Proceedings of the Royal Society B: Biological Sciences.

[bib259] Thomsen EK, Koimbu G, Pulford J, Jamea-Maiasa S, Ura Y, Keven JB, Siba PM, Mueller I, Hetzel MW, Reimer LJ (2017). Mosquito behavior change after distribution of bednets results in decreased protection against malaria exposure. The Journal of Infectious Diseases.

[bib260] Thomson RCM (1938). The reactions of mosquitoes to temperature and humidity. Bulletin of Entomological Research.

[bib261] Tjaden NB, Thomas SM, Fischer D, Beierkuhnlein C (2013). Extrinsic incubation period of dengue: knowledge, backlog, and applications of temperature dependence. PLOS Neglected Tropical Diseases.

[bib262] Tobin PC, Gray DR, Liebhold AM (2014). Supraoptimal temperatures influence the range dynamics of a non-native insect. Diversity and Distributions.

[bib263] Touré YT, Dolo G, Petrarca V, Traoré SF, Bouaré M, Dao A, Carnahan J, Taylor CE (1998). Mark-release-recapture experiments with anopheles gambiae s.l. in Banambani Village, Mali, to determine population size and structure. Medical and Veterinary Entomology.

[bib264] Umina PA, Weeks AR, Kearney MR, McKechnie SW, Hoffmann AA (2005). A rapid shift in a classic clinal pattern in *Drosophila* reflecting climate change. Science.

[bib265] Urban MC, Bocedi G, Hendry AP, Mihoub JB, Pe'er G, Singer A, Bridle JR, Crozier LG, De Meester L, Godsoe W, Gonzalez A, Hellmann JJ, Holt RD, Huth A, Johst K, Krug CB, Leadley PW, Palmer SC, Pantel JH, Schmitz A, Zollner PA, Travis JM (2016). Improving the forecast for biodiversity under climate change. Science.

[bib266] Urbanski J, Mogi M, O'Donnell D, DeCotiis M, Toma T, Armbruster P (2012). Rapid adaptive evolution of photoperiodic response during invasion and range expansion across a climatic gradient. The American Naturalist.

[bib267] Vasseur DA, DeLong JP, Gilbert B, Greig HS, Harley CD, McCann KS, Savage V, Tunney TD, O'Connor MI (2014). Increased temperature variation poses a greater risk to species than climate warming. Proceedings of the Royal Society B: Biological Sciences.

[bib268] Verhulst NO, Brendle A, Blanckenhorn WU, Mathis A (2020). Thermal preferences of subtropical *Aedes aegypti* and temperate *Ae. japonicus* mosquitoes. Journal of Thermal Biology.

[bib269] Via S, Gomulkiewicz R, De Jong G, Scheiner SM, Schlichting CD, Van Tienderen PH (1995). Adaptive phenotypic plasticity: consensus and controversy. Trends in Ecology & Evolution.

[bib270] Voorham J (2002). Intra-population plasticity of anopheles Darlingi's (Diptera, Culicidae) biting activity patterns in the state of Amapá, Brazil. Revista De Saúde Pública.

[bib271] Vorhees AS, Gray EM, Bradley TJ (2013). Thermal resistance and performance correlate with climate in populations of a widespread mosquito. Physiological and Biochemical Zoology.

[bib272] Waldock J, Chandra NL, Lelieveld J, Proestos Y, Michael E, Christophides G, Parham PE (2013). The role of environmental variables on *Aedes albopictus* biology and chikungunya epidemiology. Pathogens and Global Health.

[bib273] Waldvogel AM, Wieser A, Schell T, Patel S, Schmidt H, Hankeln T, Feldmeyer B, Pfenninger M (2018). The genomic footprint of climate adaptation in *chironomus riparius*. Molecular Ecology.

[bib274] Waldvogel AM, Feldmeyer B, Rolshausen G, Exposito-Alonso M, Rellstab C, Kofler R, Mock T, Schmid K, Schmitt I, Bataillon T, Savolainen O, Bergland A, Flatt T, Guillaume F, Pfenninger M (2020). Evolutionary genomics can improve prediction of species' responses to climate change. Evolution Letters.

[bib275] Wang G, Gordon TN, Rainwater S (2008). Maximum voluntary temperature of insect larvae reveals differences in their thermal biology. Journal of Thermal Biology.

[bib276] West-Eberhard MJ (2003). Developmental Plasticity and Evolution.

[bib277] Whitman D, Agrawal A, Whitman D, Ananthakrishnan T (2009). What is Phenotypic Plasticity and Why is it Important?. Phenotypic Plasticity of Insects.

[bib278] Willi Y, Hoffmann AA (2009). Demographic factors and genetic variation influence population persistence under environmental change. Journal of Evolutionary Biology.

[bib279] Winokur OC, Main BJ, Nicholson J, Barker CM (2020). Impact of temperature on the extrinsic incubation period of zika virus in aedes aegypti. PLOS Neglected Tropical Diseases.

[bib280] World Health Organization (2014). A Global Brief on Vector-Borne Diseases.

[bib281] World Health Organization (2018). Global Vector Control Response 2017- 2030.

[bib282] Yamana TK, Bomblies A, Eltahir EAB (2016). Climate change unlikely to increase malaria burden in West Africa. Nature Climate Change.

[bib283] Yamana TK, Eltahir EA (2013). Incorporating the effects of humidity in a mechanistic model of *anopheles gambiae* mosquito population dynamics in the Sahel region of Africa. Parasites & Vectors.

[bib284] Yang HM, Macoris ML, Galvani KC, Andrighetti MT, Wanderley DM (2009). Assessing the effects of temperature on the population of *Aedes aegypti*, the vector of dengue. Epidemiology and Infection.

[bib285] Yaro AS, Traoré AI, Huestis DL, Adamou A, Timbiné S, Kassogué Y, Diallo M, Dao A, Traoré SF, Lehmann T (2012). Dry season reproductive depression of *Anopheles gambiae* in the Sahel. Journal of Insect Physiology.

